# Histidine–Copper Site Variability in UiO-66: Monitoring Synthetic Intricacy with EPR Spectroscopy

**DOI:** 10.1021/acs.chemmater.5c01005

**Published:** 2025-09-13

**Authors:** Erlend Aunan, Isabelle Gerz, Karl P. Lillerud, Serena DeBeer, Unni Olsbye

**Affiliations:** † Centre for Materials Science and Nanotechnology, Department of Chemistry, 6305 University of Oslo, Sem Sælandsvei 26, N-0315 Oslo, Norway; ‡ Department of Inorganic Spectroscopy, 28313 Max Planck Institute for Chemical Energy Conversion, Stiftstraße 34-36, 45470 Mülheim an der Ruhr, Germany

## Abstract

Bioinspired design of catalysts aims to harness natural motifs observed in enzymatic systems to develop more efficient catalysts for industrial applications. One particularly inspiring group of enzymes—monooxygenases—demonstrate the highly efficient partial oxidations of substrates, such as the conversion of methane to methanol, a process that is highly sought after in industrial chemistry. In this study, we explore the use of zirconium-based metal–organic framework UiO-66, synthesized with open zirconium sites, to support copper–histidine complexes of varying geometries and speciation. Through the application of EPR spectroscopy, we identified three distinct copper species within the framework. The mole fractions of these copper species varied depending on the histidine loading, suggesting a tunable system with potential implications for catalytic performance. Without histidine loading, copper was found to bind scarcely to the defective site. With histidine, however, copper retention was improved. Two different species formed, of which one resembles the pMMO Cu_B_ site. Our findings lay the groundwork for further exploration and development of advanced catalysts using bioinspired design and cutting-edge MOF-characterization techniques.

## Graphical abstract

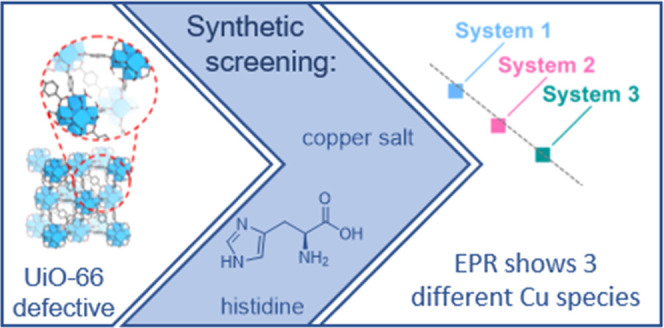

## Introduction

Certain enzymatic systems possess the remarkable ability to cleave strong C–H bonds by using copper-containing active sites. In particular, lytic polysaccharide monooxygenase (LPMO) and particulate methane monooxygenase (pMMO) have garnered significant attention in recent studies.^1–8^ LPMOs and pMMOs can activate the C–H bonds in methane and recalcitrant polysaccharides, respectively. Their ability to perform these challenging chemical conversions has inspired chemists to design a new generation of heterogeneous catalysts for methane valorization.^9–12^ In the case of pMMO, three copper-binding sites have been identified using cryo-electron microscopy,^3^ referred to as the Cu_B_ site, the Cu_C_ site, and the bis-His site. However, identifying the active site for methane activation remains both challenging and controversial,^13–16^ due in part to the enzyme’s transmembranic nature, which complicates isolation and characterization.^17–19^ LPMO on the other hand, being a smaller enzyme and more easily characterized, exhibits a single copper site that bears a resemblance to the pMMO Cu_B_ site (Figure 1B and Figure 1D). Both LPMO and pMMO Cu_B_ sites utilize an N-terminal histidine residue to establish chelation between the α- and π-nitrogens. Another histidine imidazole ring coordinates with copper, forming a distinctive T-shaped coordination motif known as a “histidine brace”. While the pMMO Cu_B_ site involves a third histidine residue to provide a 4-fold coordination, the LPMO active site remains three-coordinated.

**Figure 1:**
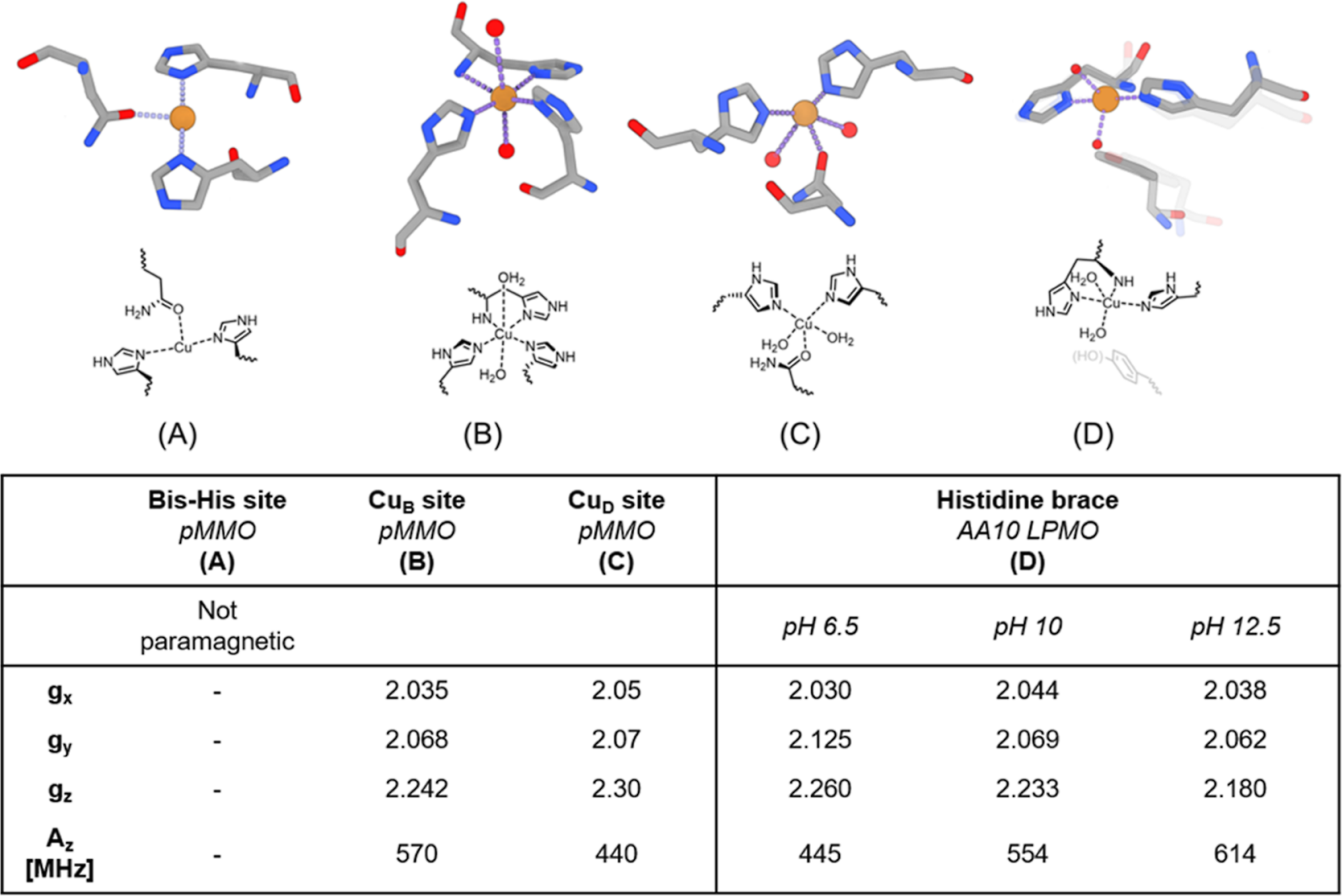
Copper sites found in the monooxygenase enzymes pMMO ((A) Bis-His site, (B) Cu_B_ site, and (C) Cu_D_-site. PDB code: 7s4h)^3^ and LPMO ((D) AA10 active site, PDB code: 4alc,^20^ and AA9 active site in transparent layer. PDB code: 4b5q).^21^ The table underneath lists literature reports of the EPR parameters for both enzymes.^14,22^

The histidine coordination to copper displayed by the monooxygenases above has been singled out as key to their activity and are sought to be imitated for novel C–H catalysts.^23,24^ However, it is obvious that there is notable diversity when it comes to the number of ligands, with either two or three histidine moieties coordinating. Also, the additional ligands can vary from different amino acids coordinating through an oxygen atom to the terminal NH_2_ of the protein backbone or water ligands. For pMMO, it has been suggested that multiple sites may contribute to the observed reactivity.^14^ Both cuprous (Figure 1A) and cupric sites (Figure 1B and C) are reported. Thus, it becomes essential to systematically replicate this diversity in the quest for a bioinspired heterogeneous catalyst capable of C–H activation. To this aim, a flexible and modular system for heterogenization is required.

Metal–organic frameworks (MOFs) are in several aspects great candidates for such a system, especially zirconium-based MOFs. Due to their high porosity and stability, they are ideal catalyst support materials when considering MOFs’ renowned ability to finely tune their chemical surface via functionalization. Since first reported in 2008,^25^ UiO-66 has been remarked for its physiochemical stability.^26,27^ UiO-66 consists of 12-connected, hexanuclear metal-oxide nodes ([Zr_6_(μ_3_-O)_4_(μ_3_-OH)_4_]^12+^) bridged by linear, ditopic terephthalate linkers (BDC). A well-established method to introduce functionality into the framework is to synthesize UiO-66 with deliberate defects known as missing-linker defects.^28–32^ At these defect sites, a terephthalate linker has been omitted from the framework and monotopic ligands such as formate or acetate are present instead as charge-compensating capping agents. Metal–organic frameworks (MOFs) have been successfully used as supports for copper species in C–H activation reactions. This approach is particularly relevant to enzyme-inspired systems such as particulate methane monooxygenase (pMMO). Notably, Yaghi and co-workers^10^ demonstrated that incorporating imidazole-bound copper into the Zr-based MOF-808 enables methane oxidation, achieving a methanol yield of 23.5 mmol MeOH per mol Cu. They propose that the reaction proceeds via a copper-oxo active site, although the process is not catalytic but rather cyclic. In contrast, Lercher and colleagues^33^ showed that installing copper-oxo-dimers on the zirconium oxide nodes of NU-1000 allows for the catalytic conversion of methane into methanol, achieving yields of 14.5 mmol MeOH/mol Cu at 1 bar and up to 34.8 mmol MeOH/mol Cu at 40 bar of methane. Similarly, we previously demonstrated that UiO-66 functionalized with histidine and copper tetrafluoroborate (UiO-66-His-Cu­(BF_4_)_2_) is active for liquid-phase oxidation of cyclohexene.^30^

It should be noted that those materials had a low histidine content (His/Zr_6_ = 0.08–0.31) and high Cu/His ratios (1.8–7.0). The catalytic motif of the materials was found to consist mainly of single Cu ions anchored to open node sites, with Cu–his as minority species.

In a follow-up study, operando characterization of the samples was performed during a redox cycle under conditions similar to literature studies of methanol partial oxidation over MOFs (vide supra), He–H_2_–He–O_2_ at 150 °C, using DFT-Assisted XAS Analysis and Multivariate Curve Resolution.^34^ The study suggested that only Cu­(II) attached to the Zr-node took part in the redox cycle, while Cu­(II) attached to histidine remained invariant within the experimental uncertainty of the method. Considering the low histidine content and high Cu/His ratios of those samples, it is of interest to explore whether higher histidine contents in UiO-66 may create Cu ligand coordination that more closely resembles that of the catalytic sites in pMMO and LPMO.

In this work, we utilize linker-defective UiO-66, with open coordination sites capped by hydroxyl/aqua ligand pairs. These pairs can serve as oxygenic ligands to copper but can also be replaced by nitrogen-containing ligands like amino acids.^10,29^ Through controlling the ligand incorporation, it may be possible to tune the copper’s coordination number, allowing for the desired level of flexibility and versatility in the catalyst system. Inspired by the enzymatic systems described above, histidine was incorporated into a linker-defective UiO-66 framework to anchor copper­(II) atoms. A systematic series of materials was synthesized, varying the degree of histidine incorporation. Due to the interplay between copper, histidine, and oxy-species present at the defect sites, a variety of possible copper coordination environments are attainable.

## Results and Discussion

The series (UiO-66-His-*X*-Cu, where *X* = 0, 2.5, 5, 7.5, 10, 15 mol equiv) was prepared by means of postsynthetic ligand exchange followed by metalation with a copper source, as illustrated in Figure 2. Both copper­(I) iodide and copper­(II) tetrafluoroborate were evaluated as potential copper sources. Regardless of the copper source used, the resulting metal site in the MOF exhibited a cupric nature, as evident by both EPR and ultraviolet–visible spectroscopy (Figures 4 and S30). The oxidation of copper­(I) upon MOF incorporation was also reported by Baek et al. for an analogous MOF-808-histidine material.^10^ In our work, copper­(I) iodide hardly incorporates into the histidine-bare MOF (UiO-66-His-0; for details, see below), whereas Cu­(BF_4_)_2_ incorporated in large amounts also in the absence of histidine ligands (see Figure S29). This finding is also supported by earlier work we reported on a similar system.^30,34^ Because of these findings, copper iodide was chosen as the copper source. The whole series was synthesized in triplicate to limit the impact of potential outliers. The synthetic procedure was adopted by the aforementioned procedure reported by Baek et al.^10^

**Figure 2:**
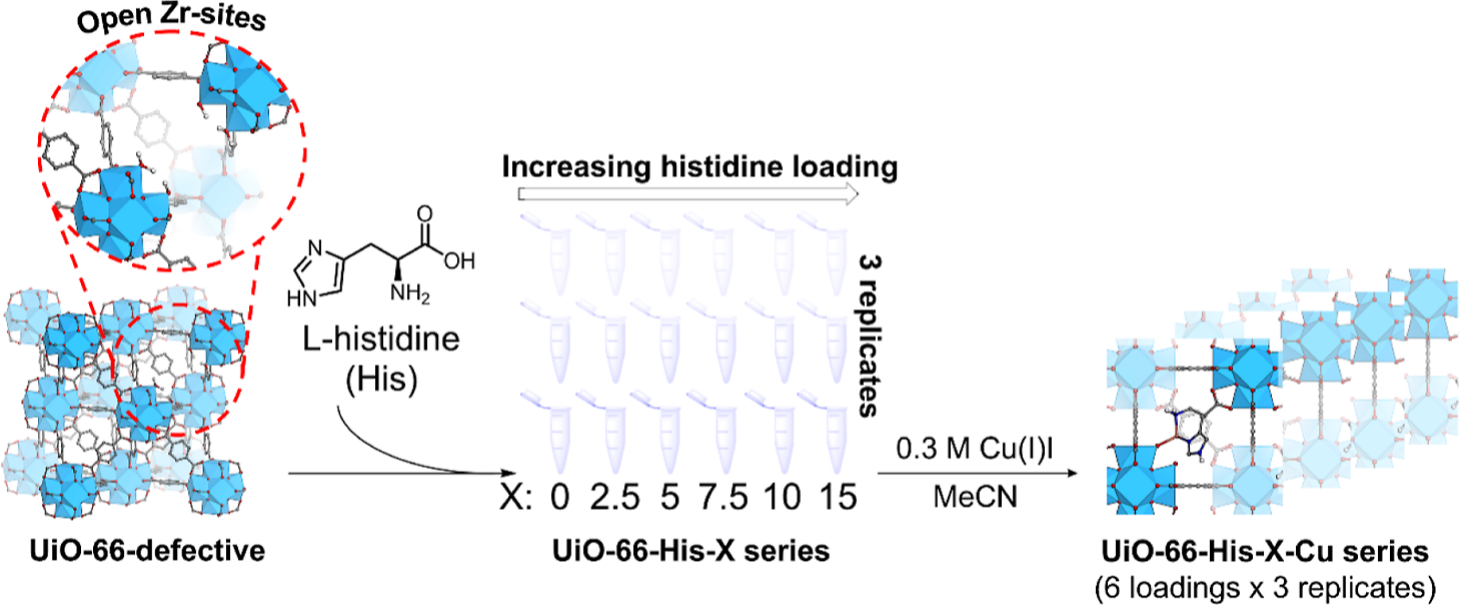
Schematic illustration of the two-step postsynthetic process to synthesize a 6 × 3 series of metalated, histidine-functionalized UiO-66.

To act as the parent material, UiO-66 was synthesized using a DMF/acetic acid-based protocol.^30^ This solvent combination leads to induced missing-linker defects, which are capped by acetate, formate, and hydroxyl/aqua ligands. These ligands originate from the usage of acetic acid, the hydrolysis of *N*,*N*-dimethylformamide (DMF), and residual water, respectively. These defect-capping agents are charge-compensating in lieu of the missing terephthalate linker, and due to their monotopic binding mode, they are relatively labile and can be replaced by other suitable ligands^29^—such as histidine.

In the first step of the postsynthetic process, the amount of histidine was varied from 0 mol equiv (zero-His experiment) to 15 mol equiv, where the molar equivalents are given relative to the amount of SBUs. The samples were washed thoroughly with water to ensure no excess histidine was present in the material, which was confirmed by testing the filtrates after each washing with the amine-detecting Kaiser’s test (Figure S28). Afterward, the degree of histidine incorporation was monitored using proton nuclear magnetic resonance spectroscopy (^1^H NMR) together with thermogravimetric analysis (TGA, Figures S20–S27). The information received from these two complementary methods is sufficient to quantify the amount of distinct organic species relative to the [Zr_6_O_4_(OH)_4_]^12+^ cluster, utilizing the method described by Sannes et al.^35^ The NMR spectra (see tabulated data in Table S2, spectra in Figures S1–S19) reveal how the incorporation of histidine into the linker-defect framework replaces the acetate and formate capping agents. The compositional analysis (Figure 3) clearly shows how histidine is incorporated proportionally to the loading. Despite the large molar excess of histidine, saturation of the missing-linker defective sites is not achieved. At the highest loading (UiO-66-His-15), approximately 55% of the available linker-defective sites are occupied by histidine.

**Figure 3:**
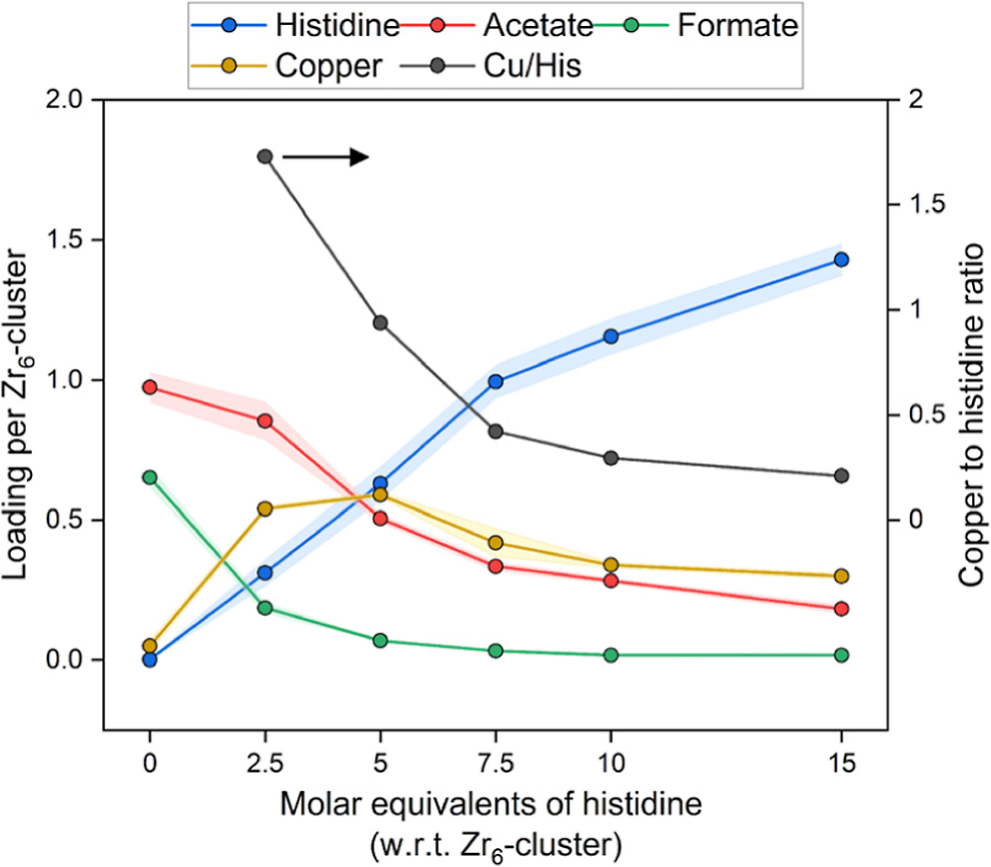
Compositional analysis of UiO-66-His-*X*-Cu as a function of the molar excess of histidine. As the available defective sites, capped by formate (green), acetate (red), and water/hydroxide (not shown, see Table S4), are increasingly exchanged with histidine (blue), the copper/histidine ratio (black) is significantly altered. Each sample was performed in triplicate, and their respective standard deviations are given as the error band following each line. Values and their errors are tabulated in Table S4.

The partial incorporation of histidine, rather than the full quantitative exchange, remains not fully understood. One plausible explanation is steric hindrance: the presence of a histidine ligand on one side of a defect site may impede the approach or binding of a second histidine on the opposite side. Additionally, the extensive washing procedures used to eliminate unbound histidine from the pores may contribute to the reduced incorporation. It is possible that the framework initially incorporated more histidine, which was then partially removed during the washing. Kaiser’s test was utilized to qualitatively monitor the amount of histidine being washed out, which is shown in Figure S28. The washing was repeated until Kaiser’s test presented close to negative for all samples, which occurred after the third washing. Notably, full incorporation of postsynthetically exchanged ligands in UiO-66 is uncommon. For instance, Shearer et al. reported that serine occupied only 45% of the linker-defective sites in UiO-66, showing a similar degree of incorporation in an analogous system.^29^ In contrast, Baek et al. investigated histidine incorporation into a different MOF (MOF-808), which also contains Zr clusters, and found that only 58% of the open Zr sites were functionalized.^10^ While the MOF topology differs, this example highlights that even for the same ligand, full site occupation is often not achieved.

For the second step, each sample was subjected to an excess of copper­(I) iodide in acetonitrile to ensure complete metalation. The samples were thoroughly washed with acetonitrile, and the washings were tested with the addition of aqueous ammonia. The washings were repeated until there was an absence of copper in the filtrate in order to ensure complete removal of excess copper from the framework. The copper-to-zirconium ratios were established using microwave plasma atomic emission spectrometry (MP-AES) on digested samples of UiO-66-His-*X*-Cu. Interestingly, the zero-His experiment (UiO-66-His-0-Cu) did incorporate a small amount of copper (0.05 ± 0.02 copper/Zr_6_), despite the lack of histidine. This copper is presumably either located at the linker-defective sites, the μ_3_-O groups at the SBU, or residing inside the pore structure of the framework as a consequence of inefficient washing. The relatively small amount of retained copper in the zero-His sample suggests that the newly introduced histidine moieties are responsible for the copper retention in the other materials. Despite using a copper­(I) source, the metal is rapidly oxidized to its copper­(II)-state over the course of the metalation, evident in the material’s color, as well as the presence of an EPR signal. Because of copper­(II)’s paramagnetic nature, the amount of histidine present in the framework could not be reassessed by ^1^H NMR after copper was incorporated. Therefore, the histidine content was assumed to remain constant across the metalation step, which is likely due to histidine’s insolubility in acetonitrile.^36^ This assumption is further reinforced by the work of Baek et al., who investigated a similar MOF-808-histidine system.^10^ Despite the presence of paramagnetic copper­(II), they were able to estimate the histidine content before and after metalation, observing only a slight decrease (from 3.5 to 3.2 His/Zr_6_). The authors also noted that the accuracy of this measurement was limited by the influence of Cu­(II), underscoring the inherent difficulty in quantifying histidine postmetalation.

Intriguingly, the copper-to-histidine ratio in the five histidine-incorporated samples decreases as the histidine loading increases, as illustrated in Figure 3. Notably, this behavior was not observed when copper­(II) tetrafluoroborate was used as the copper source instead of copper­(I) iodide (Figure S29). There, the copper increases colinearly with the histidine at low–medium loadings and plateaus at higher loadings. When the samples are metalated using copper­(I) iodide, the copper incorporation is nonlinear as a function of histidine loading (Figure 3). This trend may implicate that the copper environment changes as a function of histidine incorporation.

X-band CW EPR spectroscopy (Figure 4) offers a selective probe of the paramagnetic copper­(II) center in the material, making it a suitable technique to investigate the effect of histidine loading on the copper site. The spectra’s appearance varied throughout the UiO-66-His-*X*-Cu series. The EPR spectra of the histidine-loaded samples were simulated together, and two copper species (System 1 and System 2) were necessary to explain the series of spectra. Their relative weights for the overall simulation changes throughout the series, but the parameters for System 1 and System 2 are equal for all five spectra (see Figure 4A). Additionally, the zero-His sample was fitted separately as it showed a distinctively different EPR signal (Figure 4B) attributed to a third copper species, System 3.

**Figure 4:**
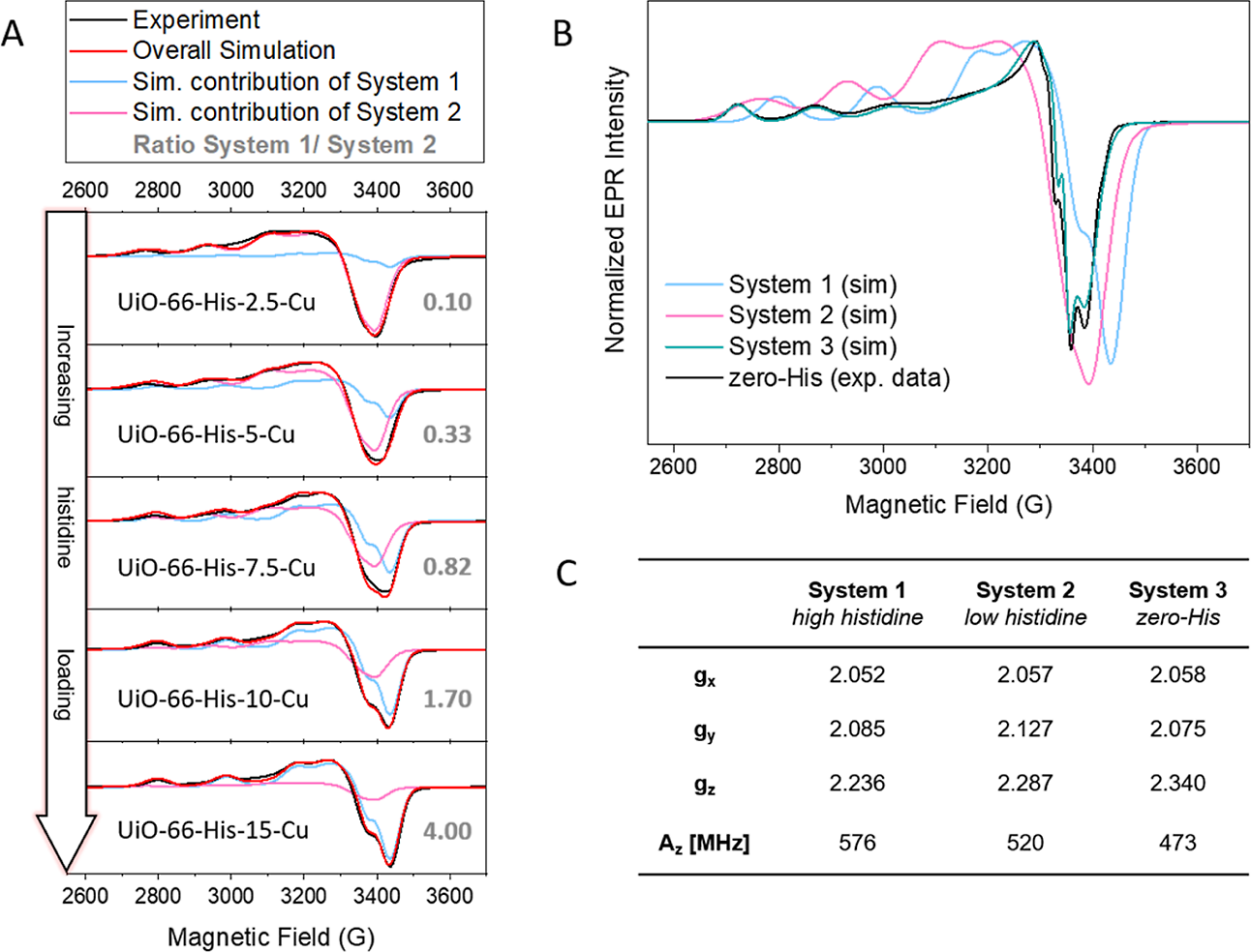
EPR analysis of the copper site in the histidine-functionalized UiO-66. (A) X-band CW EPR spectra (30 K, neat powder) of the series of MOFs. The spectra were simulated with two species (System 1 and System 2) of varying relative weights. The System 1/System 2 ratio is given for each spectrum. (B) X-band CW EPR spectrum of the zero-his and its simulation (System 3) overlaid with the two simulated species from the histidine-loaded samples (Systems 1 and 2). (C) Selected parameters used for the simulation of the spectra.

For the histidine-loaded samples, a progressive change in the mole fractions of the two species is observed (Figure 4A). The high loadings are dominated by a relatively axial species (System 1), while a more rhombic species (System 2) makes up the majority of the EPR signal at lower loadings. The EPR parameters of the axial species System 1 (Figure 4C) compare well to those of the Cu_B_ site of pMMO reported by Jodts et al., who reported *g* = [2.24, 2.07, 2.035], and a ^63^Cu hyperfine splitting *A*_∥_ = 580 MHz.^37^ The pMMO Cu_B_ site is formed by three histidines, and a 1:3 Cu/His ratio may be achieved locally for the system, despite the overall observed Cu/His ratio for UiO-66-His-15-Cu of 0.2 suggesting more histidine ligands. For one, it is not given that the metalation (or any functionalization) affects the entirety of the sites in a MOF due to diffusion or unavailability of sites.^38^ Second, due to geometric restrictions arising from the topology of the MOF, five histidines pointing toward the same copper atom are unlikely. Suggesting a coordination motif for System 2 is more challenging and will be discussed later (vide infra). The spectrum of the zero-His sample was fitted with System 3, a rather axial species (Figure 4). Shan et al. have reported the CW EPR spectrum of UiO-66-Cu, where the copper is bound directly to the linker-defective site, having an *A*_∥_ = 470 MHz and a *g*_∥_ = 2.34.^39^

This is in excellent agreement with the parameters found for System 3. Thus, the copper species in System 3 is likely directly bound to the zirconium-oxo cluster. Comparable EPR parameters have also been reported for hydrated Cu zeolites,^40^ which are microporous systems with properties comparable to those of MOFs.

The parameters extracted for the three species can be analyzed to suggest the nature of their ligands, as not only histidine can bind to copper but there are also several oxygen-based sites on the zirconium cluster itself that can act as a binding site. Peisach and Blumberg mapped out the effect of nitrogen vs oxygen ligands on the parallel component of the EPR spectrum.^41^ Considering four coordinating atoms, they found that they could attribute areas of an A_∥_ over g_∥_ plot to each composition possible (from four coordinating nitrogens in the top left corner, over ligands with a mixed composition, to those exclusively having ligands coordinating through oxygen atoms found in the bottom left corner). The charge of the complexes (+2 to −2) elongates the areas so that they overlap. The concept was later expanded to investigate copper sites in zeolite.^42^

Therefore, the parameters of the parallel component of the two copper species found in the histidine-loaded MOFs and the copper species found in the zero-His sample were marked on a Peisach-Blumberg plot (Figure 5). The number of coordinating atoms is not known for our systems; therefore, the areas mapped out specifically for four-coordinate species are not necessarily accurate. Godiksen et al. found that hydrated zeolites (where all coordinating atoms are oxygens) showed an offset from the trendline toward the upper right corner, which was attributed to the higher coordination number.^42^ Consequently, the Peisach-Blumberg plot assigns neither coordination number nor ligand identity nor charge. Instead, it offers a comparative indication of the coordination sphere, taking all three factors into account. Assuming that all species in this study are either neutral or positively charged copper sites, the copper coordination in System 1 is dominated by nitrogen ligands. This, again, is in good agreement with a copper-site resembling the Cu_B_ site in pMMO.

**Figure 5:**
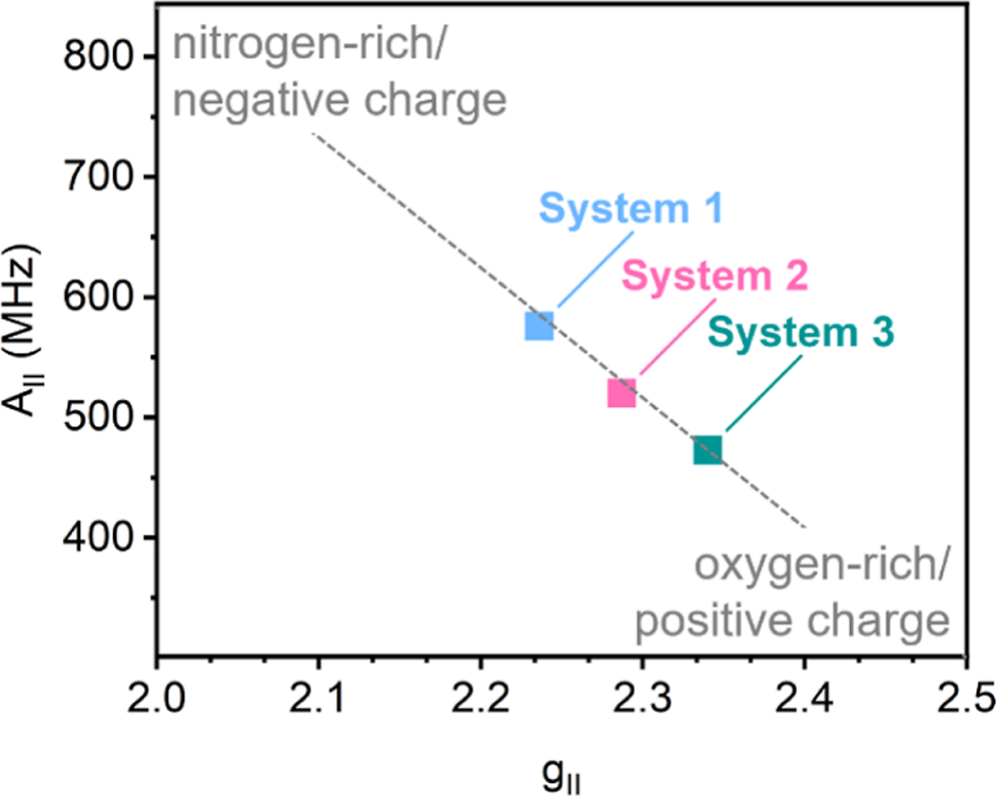
Peisach–Blumberg plot^41^ of Systems 1–3.

For System 2, however, the placement between the other two species in the Peisach-Blumberg plot suggests that the site may have one or several oxygen ligands. A possible coordination motif is the histidine–copper site we described previously,^30^ in a similar system. There, copper is bound to both histidine through the amino group and the imidazole ring and completes its coordination shell with oxygen ligands from the Zr_6_-node of the MOF and an OH^–^ ligand.

Valverde et al. found similar parameters for the parallel component of MOF-808@His-Cu (*g*_∥_ = 2.274; *A*_∥_ = 525 MHz) and attribute these values to a copper site coordinated by two nitrogen and two oxygen sites.^43,44^ This is in line with the closely related system MOF-808-His-Cu reported by Baek et al., which was also described to have a 2N2O coordination environment.^10^ MOF-808 and UiO-66 have different topologies (spn vs fcu), which translates into different coordination geometries for the histidine-copper sites. Consequently, the EPR signal of System 2 is more rhombic than the axial signal of MOF-808@His-Cu. It is also worth noting that literature reports show that at neutral pH (= 6.5), association of water ligands causes a rhombic distortion of the active site in LPMOs, creating a copper environment comprised of two histidines and two water molecules.^22,45^ We therefore propose that System 2 is a cupric copper site bound by histidine and water or other oxygen ligands found in the framework.

The copper site found in the zero-His sample (UiO-66-His-0-Cu, see EPR spectrum in Figure 4B and simulation parameters in Figure 4C) is located even farther toward an oxygen-rich ligand-set in the Peisach–Blumberg plot. This is consistent with being bound to the defective site of the cluster, coordinated by oxygen atoms of the cluster and completing its coordination shell by water or other capping agents.

## Conclusions

Three distinct copper sites could be generated within the same framework by functionalization of missing-linker defects in MOFs with histidine. One copper site exhibits an EPR signal that is in good agreement with the Cu_B_-site in pMMO, underlining the potential of highly tunable microporous systems to create enzyme-like environments. However, careful screening of the synthetic protocol is necessary as the tunability comes with a complex interplay of the two functionalization steps. The synthesis scales neither quantitatively nor qualitatively with the histidine incorporation, meaning that a higher histidine loading does not retain more copper and that the dominant type of copper site is histidine-loading dependent.

By choice of the copper source (CuI vs Cu­[BF_4_]_2_), it is possible to favor either node-bound or histidine-bound copper species. For copper iodide, a strong preference for histidine-bound species is observed. The dominant species depends on the histidine loading, meaning that the synthesis can be tuned to yield either species or create a mixture of both.

The different coordination environments not only showcase the variability of the synthetic approach but also yield copper sites of interest for catalysis. Recently, the CuB site of pMMO, to which System 1 showed a resemblance, was connected to peroxide production.^46^ Meanwhile, the node-bound copper site (System 3) has shown to be a versatile catalyst, both in C–H activation and ammonia production from nitrate.^30,39^ System 2 may also have potential as a catalytic site, given that similar g_∥_ and A_∥_ were found for MOF-808@His-Cu, which is closely related to the MOF-808-His-Cu reported by Baek et al.^10^ The latter has both copper­(I) and copper­(II) sites and is an active catalyst for methane to methanol conversion.

EPR spectroscopy proved to be a suitable tool to monitor systematic amino acid loading dependencies on the copper site. The method presented herein for producing a series of materials with varying ligand loadings and analyzing the effect on the resulting metal coordination site by CW X-band EPR spectroscopy is applicable to the development of synthesis protocols for other EPR-active metal centers.

## Experimental Section

### Chemicals

l-Histidine (cell culture grade) and zirconyl chloride (98%) were purchased from Fisher Scientific. Acetic acid (glacial), acetonitrile (HPLC grade), acetone (technical grade), ethanol (absolute), sulfuric acid (98%), hydrogen peroxide (30%), and *N*,*N*-dimethylformamide (EMPLURA) were purchased from VWR. Copper­(I) iodide was purchased from Merck (Sigma-Aldrich). All chemicals were used as received without further purification.

### Instrumentation

Powder X-ray diffraction was performed with a Bruker D8 Discover diffractometer using Cu Kα1 radiation selected by a Ge(111) Johanssen monochromator. Thermogravimetric analysis was performed using a NETZSCH STA 449 F3 Jupiter, ramping from 30 to 900 °C with a 10 K min^–1^ ramping rate. The samples were under a stream of synthetic air, consisting of a 40 mL min^–1^ flow rate of N_2_ and 20 mL·min^–1^ flow rate of O_2_ and 40 mL min^–1^ flow rate of N_2_ as protective gas. Nuclear magnetic resonance was performed using a Bruker AVII 600, operating at 600 MHz (^1^H). MOF samples were digested prior to the analysis. Approximately 20 mg of MOF was calcined at 200 °C overnight, before being digested in 1 M NaOD solution in D_2_O for 10 min. The mother liquor was then separated from precipitated Zr­(OD)_4_ by centrifugation and then analyzed by ^1^H NMR spectroscopy. Elemental analysis was performed using an Agilent 4100 Microwave Plasma Atomic Emission Spectrometer (MP-AES). A calibration curve containing copper and zirconium in 1% aqueous H_2_SO_4_, covering the range from 10 to 100 ppm Zr and 0.1 to 10 ppm of Cu was used. Samples (7.5–12.5 mg) were digested 1 day prior to analysis in 500 μL sulfuric acid (conc.) in glass vials at 110 °C. Then, after an addition of 100 μL H_2_O_2_ *(Caution: Exothermic!)*, the clear solution was diluted with water to 50 mL before analysis.

### Synthesis

#### UiO-66-Defective

Zirconyl chloride (40.0 g, 0.124 mol, 1.00 equiv) was dissolved in DMF (260 mL, 27 equiv) at room temperature. After stirring for ca. 5 min, a clear solution was obtained. Acetic acid (glacial, 260 mL, 37 equiv) was then added while stirring, and the solution was transferred to a suitable round-bottom flask containing terephthalic acid (31.7 g, 0.191 mol, 1.5 equiv). The flask was fitted with a condenser with a calcium chloride drying tube, and the suspended mixture was heated to 90 °C. After it was stirred overnight, the reaction mixture was filtered while still hot. The filter cake was resuspended in DMF (200 mL) in a round-bottom flask and stirred at 90 °C for 1 h before refiltering. The MOF was then transferred to a 1000 mL HDPE container together with 800 mL H_2_O, and continuously agitated for 24 h, while replacing the water twice. The next day, the filtration was repeated, and the MOF was again transferred to a 1000 mL HDPE container together with acetone and agitated overnight. Finally, the MOF was filtered and dried, first at 75 °C for 2 h and then at 150 °C overnight, yielding UiO-66-defective (26.5 g, 84% yield, based on the observed molar weight of the product).

#### Approximated Chemical Formula

Zr_6_O_4_(OH)_4_(BDC)_4.0_(OAc)_2.1_(H2O)_1.9_(OH)_1.9_ with 33% missing linker defectivity. The molecular mass of the material was 1529 g mol^–1^, as measured by thermogravimetry.

#### UiO-66-Histidine Series

For each sample (= histidine loading), the experiment was performed in triplicate.

UiO-66-defective (300 mg, 0.20 mmol, 1.0 equiv) was added to a 15 mL HDPE Falcon tube containing histidine (0–15 equiv., see Table S1), followed by water (5.0 mL). The tubes were capped, placed in on a rack, and inserted into an oven at 60 °C for 18 h. The following day, they were centrifuged while still warm, and the mother liquor was poured off prior to histidine crystallizing. Water (10 mL, room temp.) was added to each tube and was continuously agitated for 10 min before being centrifuged. An overnight washing step was also included. To ensure efficient washing of the histidine-inserted materials, Kaiser’s test was used on the filtrate solutions to ensure the absence of amine acids. For this, approximately 10 mg of ninhydrin was dissolved in 10 mL of methanol, and three drops of the clear ninhydrin solution was added to 1 mL of the filtrate in a test tube. The solution was heated to a boil using a heat gun, and the presence of amino acids was assayed visually based on the color of the solution. If no color appeared, the filtrate was determined to be free of histidine and the washing procedure was stopped (see Figure S2). When water washings were finished, solvent exchange using ethanol (2 × 10 mL) was performed, before drying at 70 °C for 4 h yielding UiO-66-His-*X* (yields are given in Table S1, and compositions are in Table S6).

#### UiO-66-His-Cu Series

UiO-66-His-*X* (225 mg) was charged into a 15 mL HDPE Falcon tube. To each tube, a solution of copper­(I) iodide (5.0 mL, 0.34 mol L^–1^) in acetonitrile was added. The tubes were capped and continuously agitated at an orbital shaker plate (IKA) for 48 h. Each tube was centrifuged, and the mother liquor was poured off and replaced with fresh acetonitrile (10 mL). This washing procedure was repeated three times and then twice with ethanol (10 mL). The samples were then gently dried at 70 °C overnight, yielding UiO-66-His-*X*-Cu (compositions are given in Table S6.).

### EPR Measurements

The MOFs were measured as neat powders (about 40 mg). The continuous-wave X-band (∼9.64 GHz) EPR spectra were measured on a Bruker ELEXSYS E500 spectrometer (Billerica, MA, USA) equipped with a SuperX microwave unit, a Bruker dual-mode cavity (ER4116DM), and an Oxford ESR 900 liquid helium continuous-flow cryostat, which held the experimental cavity at a temperature of 30 K. Spectra were collected with a field modulation amplitude of 7.460 G at a frequency of 100 kHz and using a 5.12 ms time constant and 81.92 ms conversion time for the collection of a 1024-point spectrum for each scan. A total of 10 scans were collected for each sample. All spectra were simulated in MATLAB R2019b with the EasySpin package (version 6.0.0-dev.50, release 2022-12-10).^47^

## Supplementary Material



## Notes

Detailed parameters of the synthetic procedure and characterization data (^1^H NMR spectroscopy, MP-AES, TGA, DR-UV/vis spectroscopy, pXRD) (PDF)
