# Supplementary material for: Histidine–Copper Site Variability in UiO-66: Monitoring Synthetic Intricacy with EPR Spectroscopy
Source: Chem Mater. 2025 Sep 13;37(19):7648–55. doi: 10.1021/acs.chemmater.5c01005 (PMC12529766; doi:10.1021/acs.chemmater.5c01005)
Supplement: Supplementary file 1 [file cm5c01005_si_001.pdf]

Supporting information for:

Histidine-Copper Site Variability in UiO 66: Monitoring Synthetic Intricacy with EPR Spectroscopy

Erlend Aunan[a§], Isabelle Gerz[b§], Karl P. Lillerud[a], Serena DeBeer[b*], Unni Olsbye[a*]

[a]Centre for Materials Science and Nanotechnology, Department of Chemistry, University of Oslo, Sem Sælandsvei 26, N-0315 Oslo, Norway

[b]Department of Inorganic Spectroscopy, Max Planck Institute for Chemical Energy Conversion, Stiftstraße 34-36, 45470 Mülheim an der Ruhr, Germany

[*] Email: serena.debeer@cec.mpg.de

[*] Email: unni.olsbye@kjemi.uio.no

[§] E.A. and I.G. contributed equally to this work

Table of Contents

Experimental design and synthesis 3
^{1}H NMR spectroscopy 4
Microwave-plasma atomic emission spectroscopy 15
Thermogravimetric analysis 16
Thermograms for Series 1: 16
Thermograms for Series 2 20
Compositional summary 20
Diffuse reflectance UV-VIS 22
Powder X-ray diffraction 23
EPR measurements 23

Experimental design and synthesis

Table S1: Experimental parameters for the synthesis of the UiO-66-His-X series 1.

Sample	Replicate	UiO-66 [g]	Histidine [g]	Histidine [mol. equiv.]	Yield [g]
**0**	1	0.2984	0.0000	0	0.293
	2	0.2999	0.0000	0	0.283
	3	0.2997	0.0000	0	0.394
**1**	1	0.3005	0.0778	2.552	0.284
	2	0.3008	0.0771	2.526	0.300
	3	0.2997	0.0769	2.529	0.308
**2**	1	0.3003	0.1544	5.067	0.297
	2	0.2997	0.1545	5.080	0.296
	3	0.3006	0.1551	5.085	0.280
**3**	1	0.3001	0.2330	7.652	0.309
	2	0.3006	0.2329	7.636	0.298
	3	0.3009	0.2333	7.641	0.306
**4**	1	0.2998	0.3107	10.214	0.314
	2	0.2998	0.3101	10.194	0.306
	3	0.3004	0.3101	10.173	0.306
**5**	1	0.2999	0.4661	15.317	0.302
	2	0.2999	0.4657	15.304	0.319
	3	0.3003	0.4652	15.267	0.313

^{1}H NMR spectroscopy

Table S2: ^{1}H NMR integrals of digested samples from the UiO-66-his-X series 1. (600 MHz, 1 M NaOD in D_2O)

Sample	Replicate	NMR integrals[a]		
		Formate[b]	Histidine[c]	Acetate[d]
**0**	1	0.163888	0.001123	0.737779
	2	0.160133	0.000912	0.716837
	3	0.161175	0.000517	0.721353
**1**	1	0.038987	0.074907	0.538919
	2	0.039212	0.080384	0.551804
	3	0.044577	0.052216	0.599386
**2**	1	0.013721	0.153254	0.321692
	2	0.015794	0.142157	0.345608
	3	0.016987	0.126253	0.349837
**3**	1	0.007419	0.20689	0.221796
	2	0.008263	0.228523	0.220108
	3	5.65E-03	0.225321	0.226375
**4**	1	0.002257	0.226432	0.170517
	2	0.002345	0.227892	0.182629
	3	0.005889	0.260462	0.172922
**5**	1	0.005887	0.295066	0.119032
	2	0.003437	0.304044	0.121525
	3	0.00161	0.313588	0.108382

a: Integrals given as ratios to terephthalate's singlet at 7.79 ppm (4H)
b: Singlet (1H) at 8.37 ppm
c: Average of singlet at 6.86 ppm (1H), dd at 2.91 ppm (1H), and dd at 2.76 ppm (1H)
d: Singlet (3H) at 1.92 ppm

An example of a typical ^{1}H NMR spectrum , as well as the used integration ranges is shown in **Figure S1**. The integration ranges were kept identical for all 18 spectra.

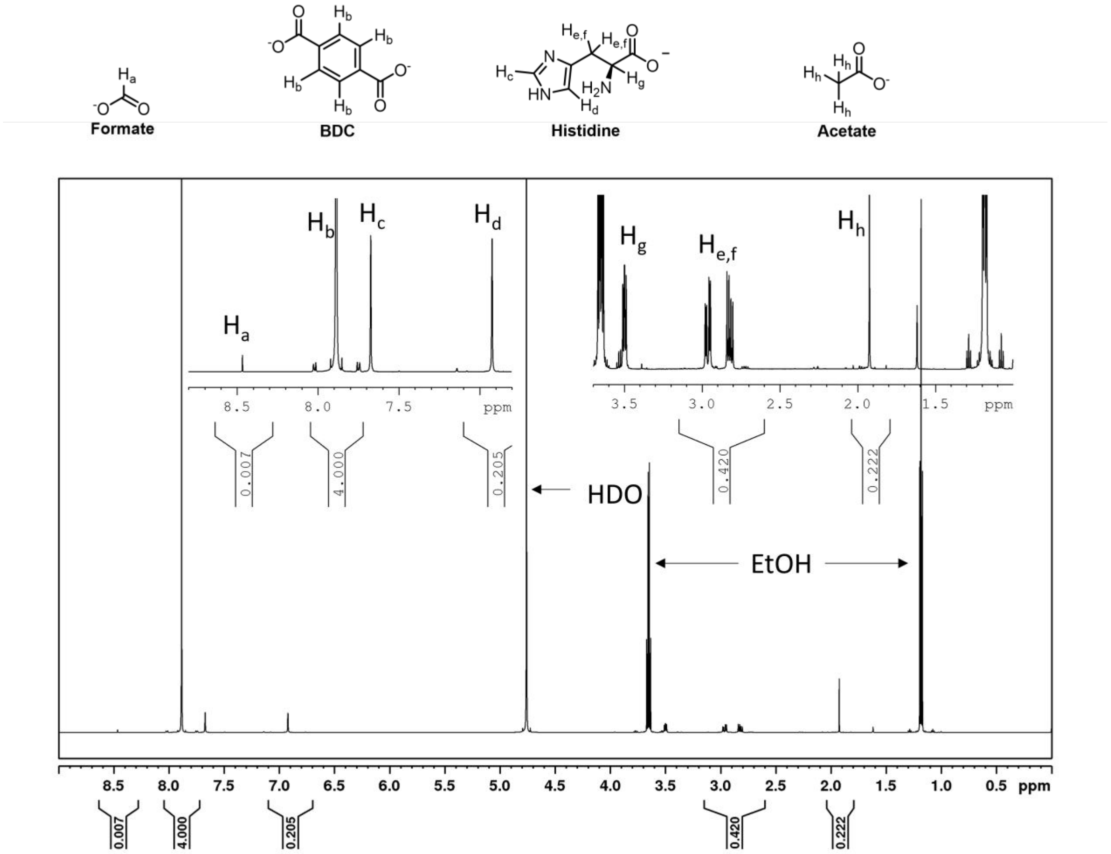

Figure S1 ^{1}H NMR spectrum (600 MHz, 1 M NaOD in D_2O) of UiO-66-his-7.5, replicate 1, with inserts highlighting the integral ranges used for all spectra in this work.

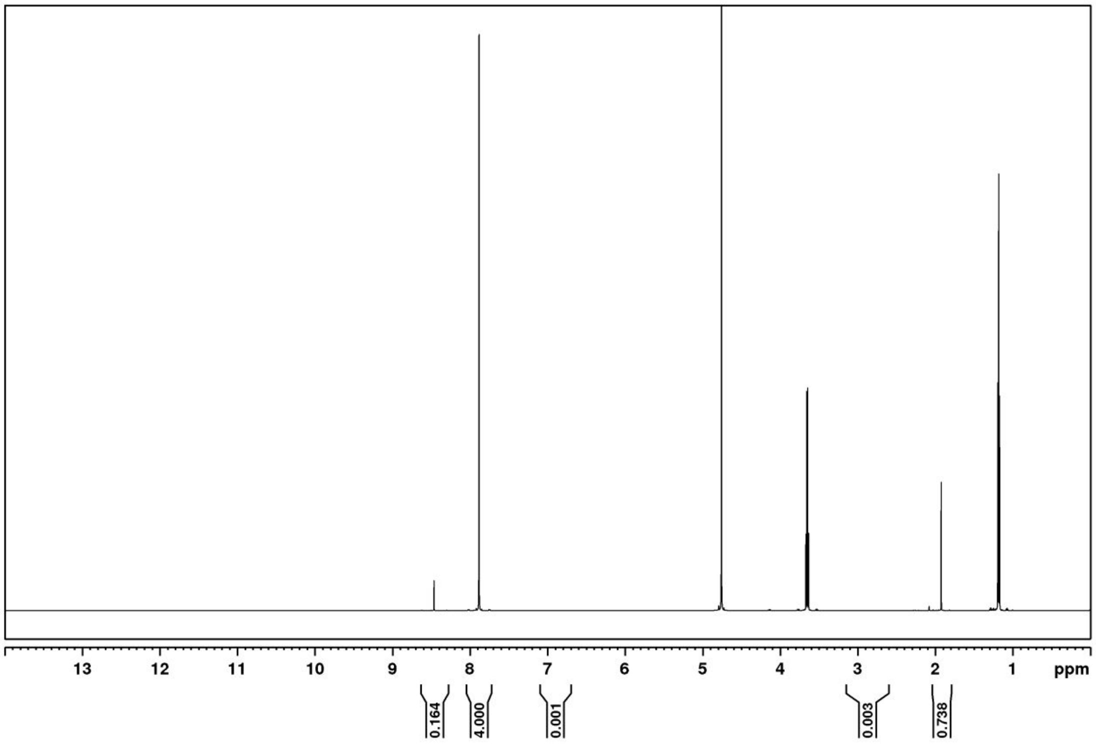

Figure S2 ^{1}H NMR spectrum (600 MHz, 1 M NaOD in D_2O) of UiO-66-his-0, replicate 1

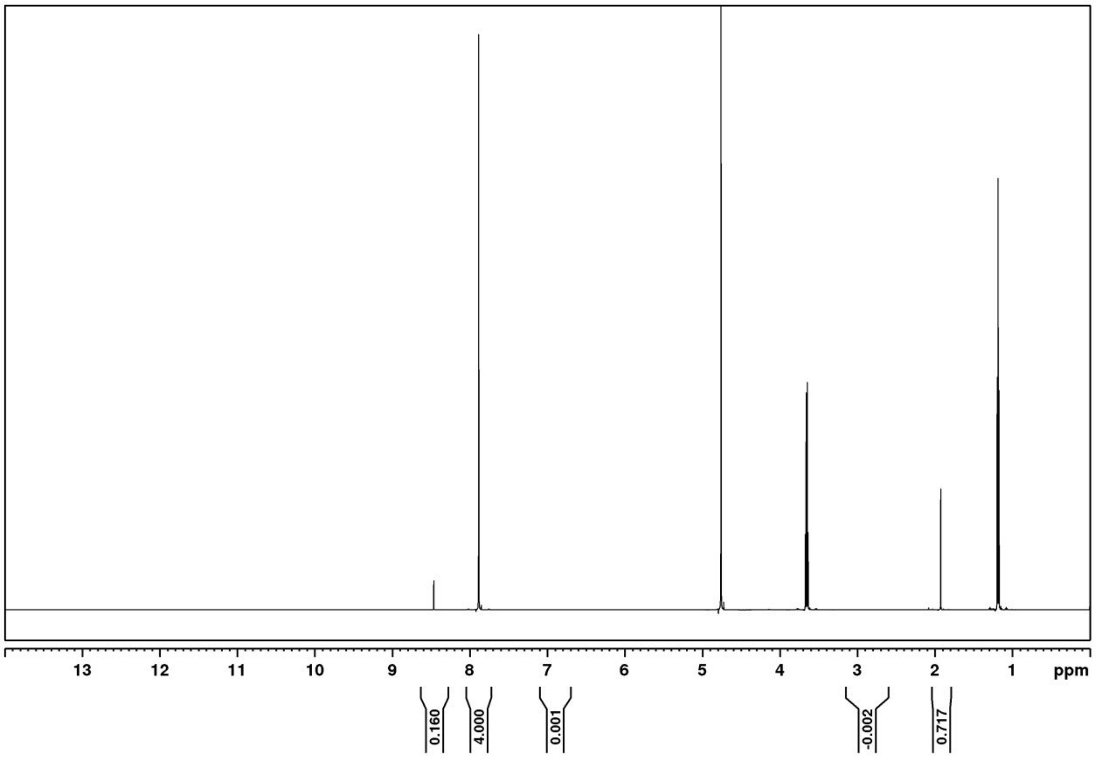

Figure S3 ^{1}H NMR spectrum (600 MHz, 1 M NaOD in D_2O) of UiO-66-his-0, replicate 2

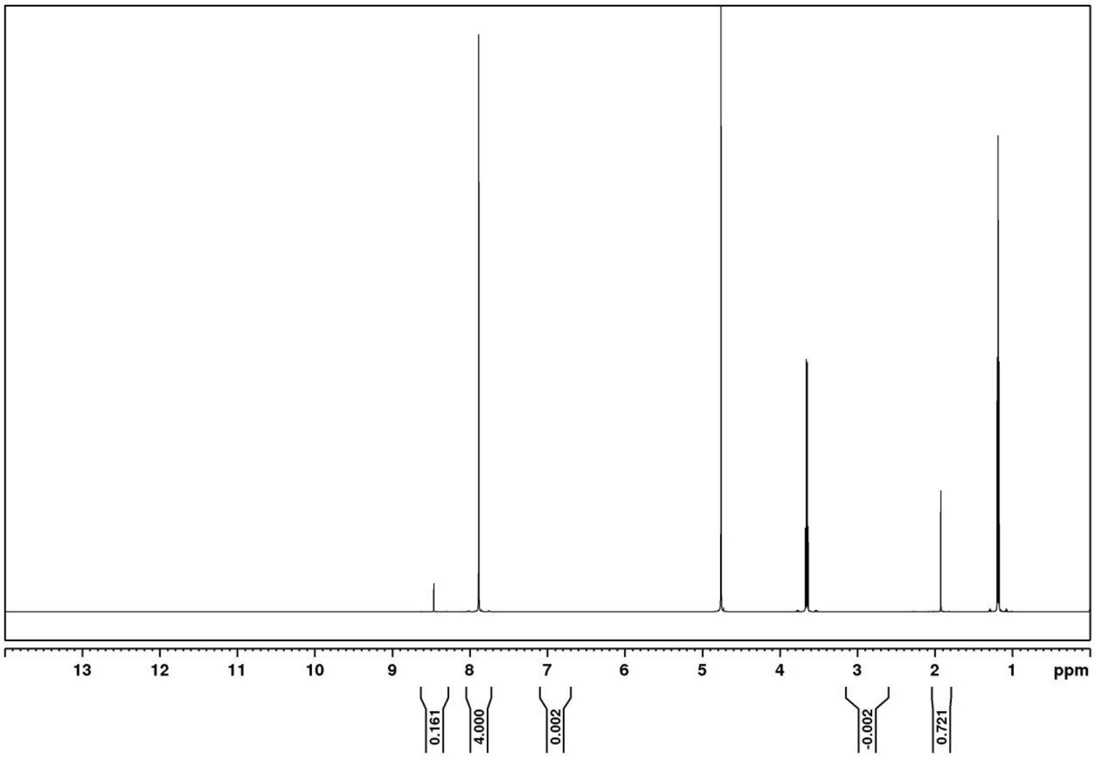

Figure S4 ^{1}H NMR spectrum (600 MHz, 1 M NaOD in D_2O) of UiO-66-his-0, replicate 3

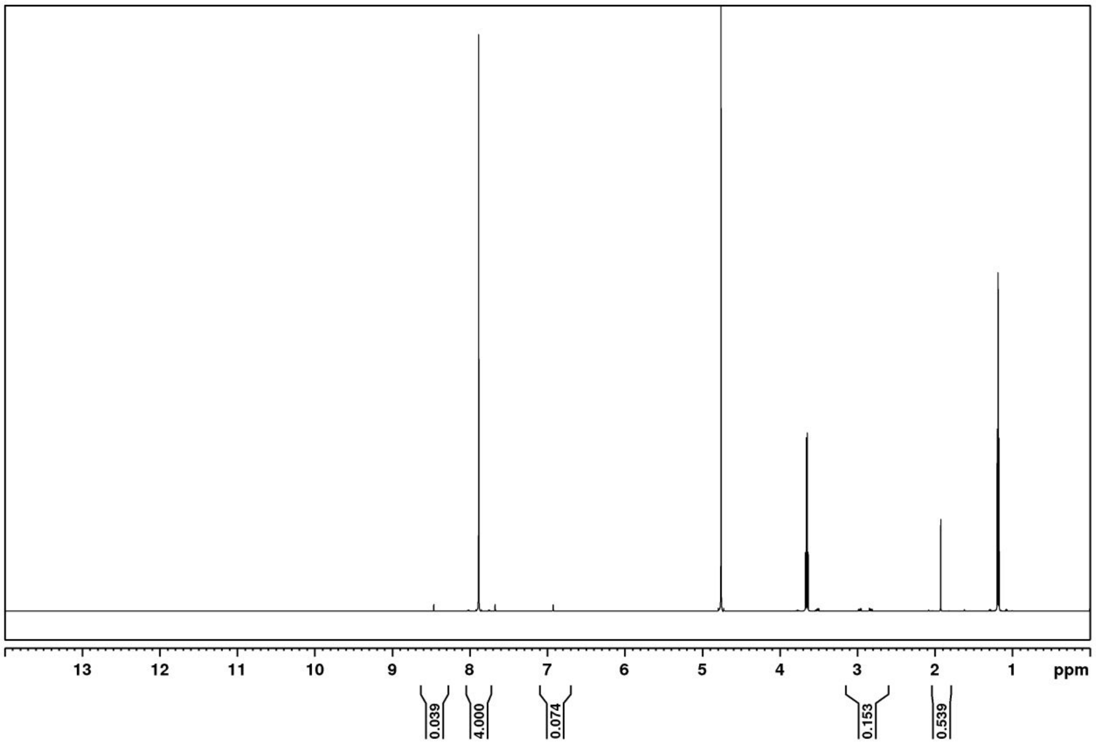

Figure S5 ^{1}H NMR spectrum (600 MHz, 1 M NaOD in D_2O) of UiO-66-his-2.5, replicate 1

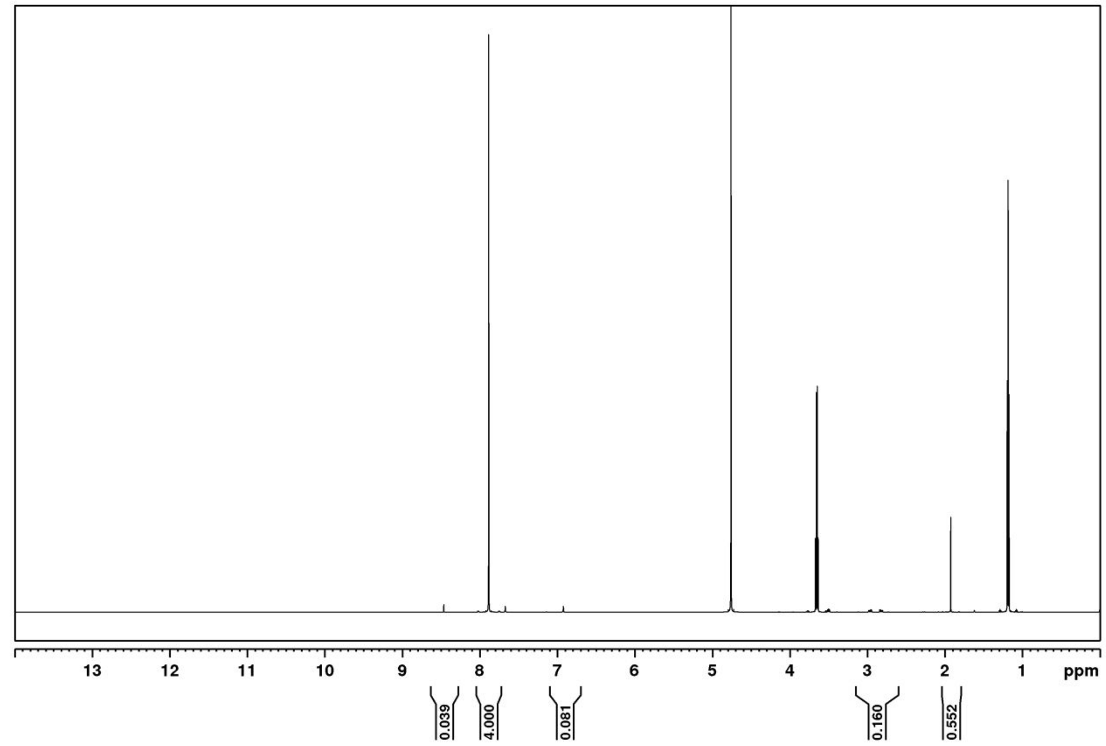

Figure S6 ^{1}H NMR spectrum (600 MHz, 1 M NaOD in D_2O) of UiO-66-his-2.5, replicate 2

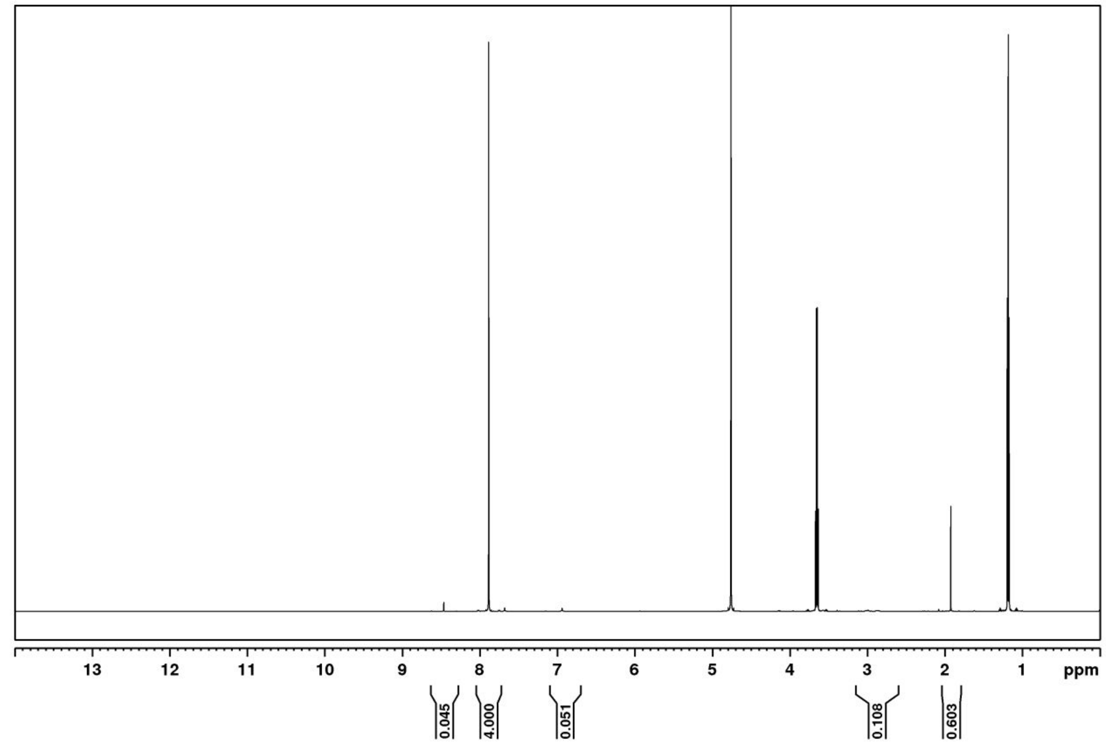

Figure S7 ^{1}H NMR spectrum (600 MHz, 1 M NaOD in D_2O) of UiO-66-his-2.5, replicate 3

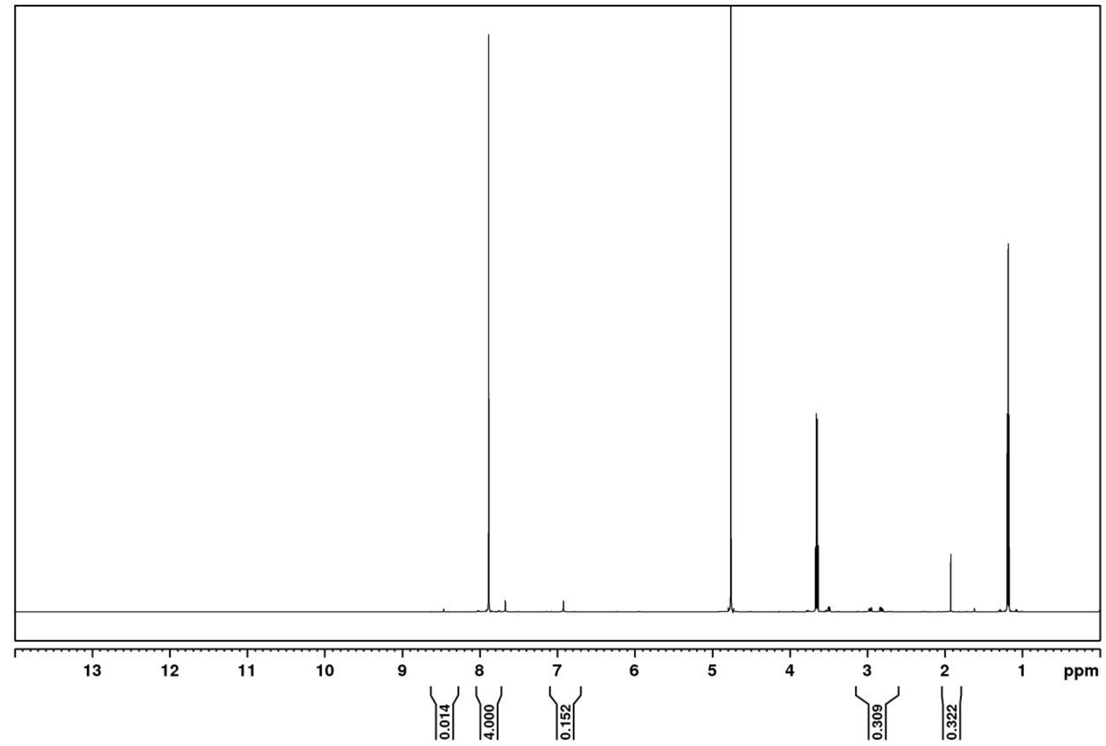

Figure S8 ^{1}H NMR spectrum (600 MHz, 1 M NaOD in D_2O) of UiO-66-his-5, replicate 1

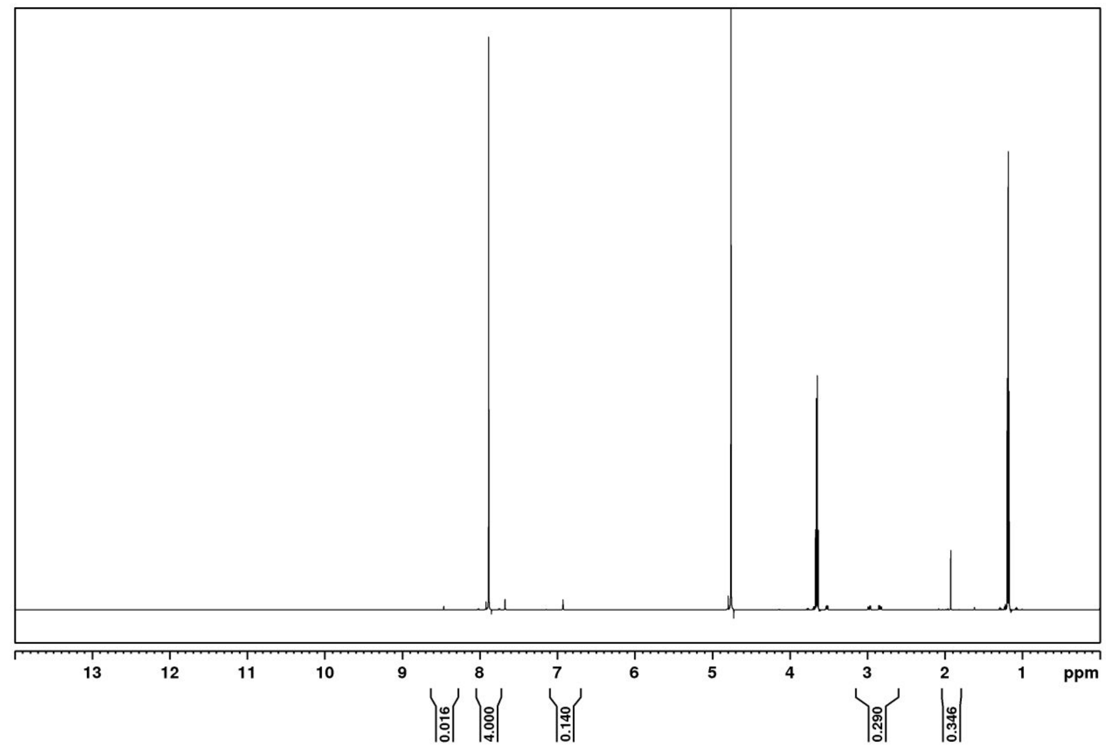

Figure S9 ^{1}H NMR spectrum (600 MHz, 1 M NaOD in D_2O) of UiO-66-his-5, replicate 2

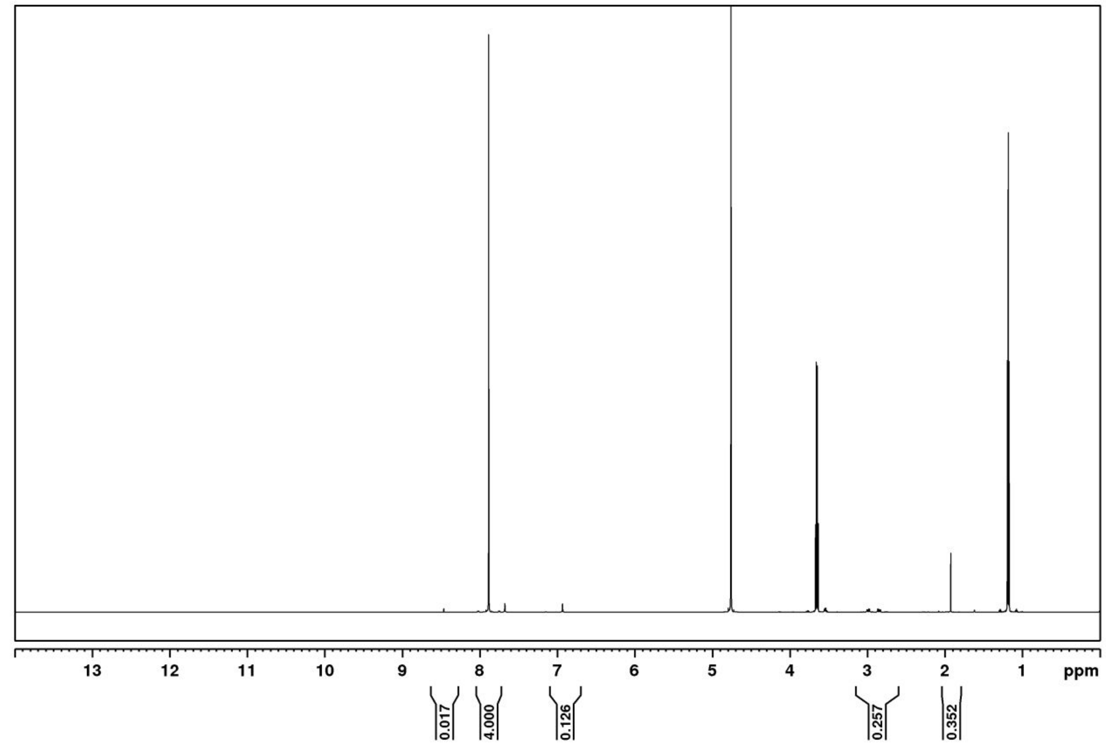

Figure S10 ^{1}H NMR spectrum (600 MHz, 1 M NaOD in D_2O) of UiO-66-his-5, replicate 3

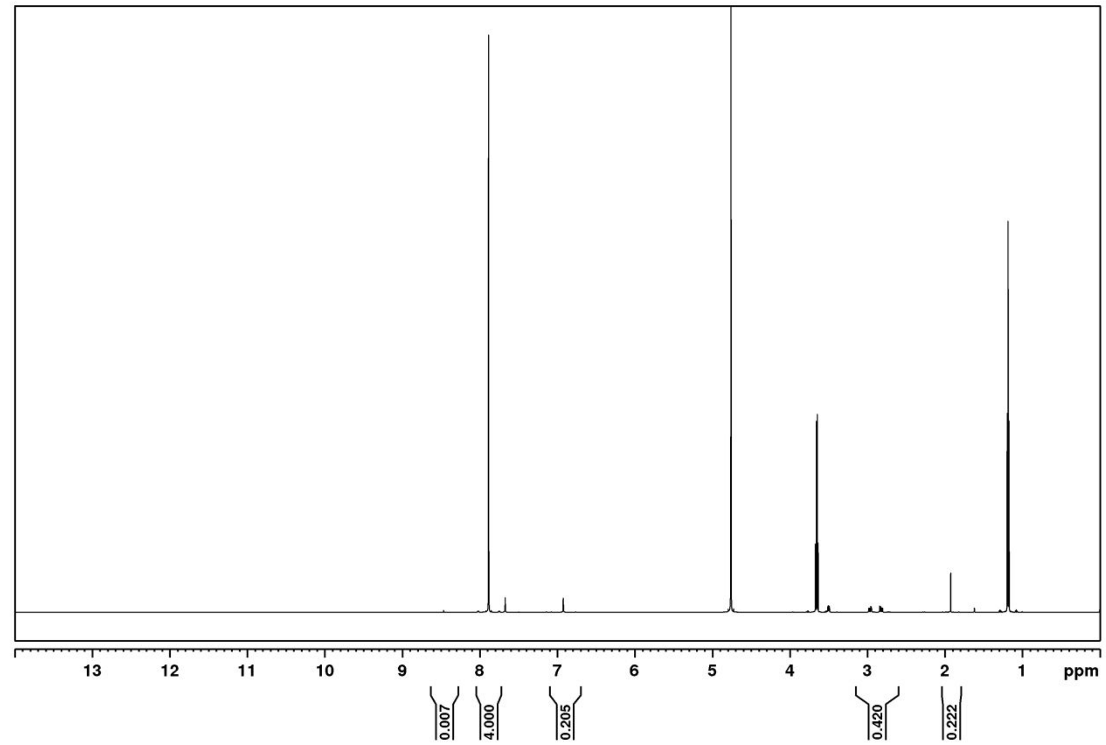

Figure S11 ^{1}H NMR spectrum (600 MHz, 1 M NaOD in D_2O) of UiO-66-his-7.5, replicate 1

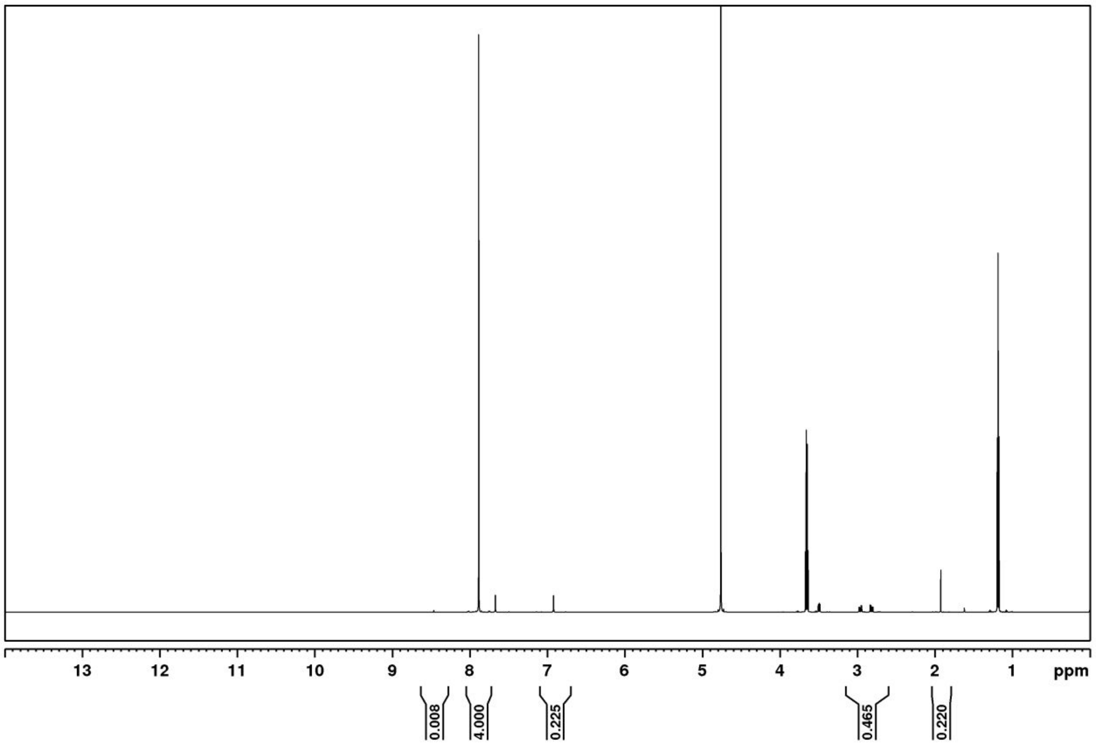

Figure S12 ^{1}H NMR spectrum (600 MHz, 1 M NaOD in D_2O) of UiO-66-his-7.5, replicate 2

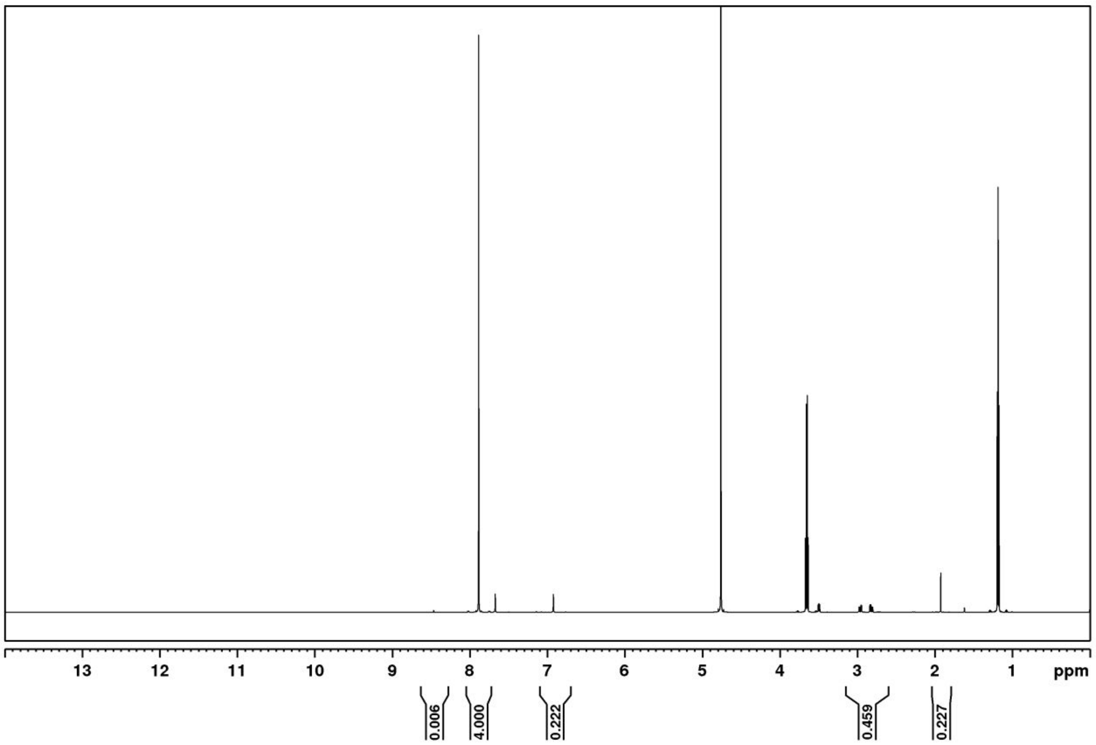

Figure S13 ^{1}H NMR spectrum (600 MHz, 1 M NaOD in D_2O) of UiO-66-his-7.5, replicate 3

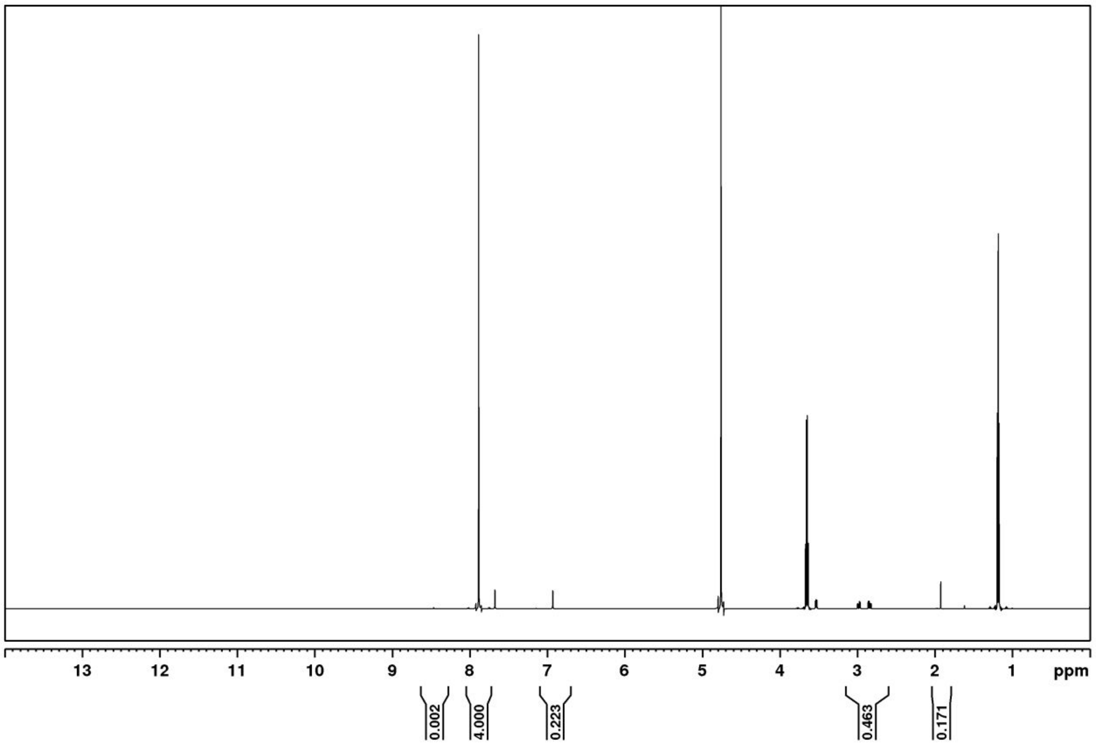

Figure S14 ^{1}H NMR spectrum (600 MHz, 1 M NaOD in D_2O) of UiO-66-his-10, replicate 1

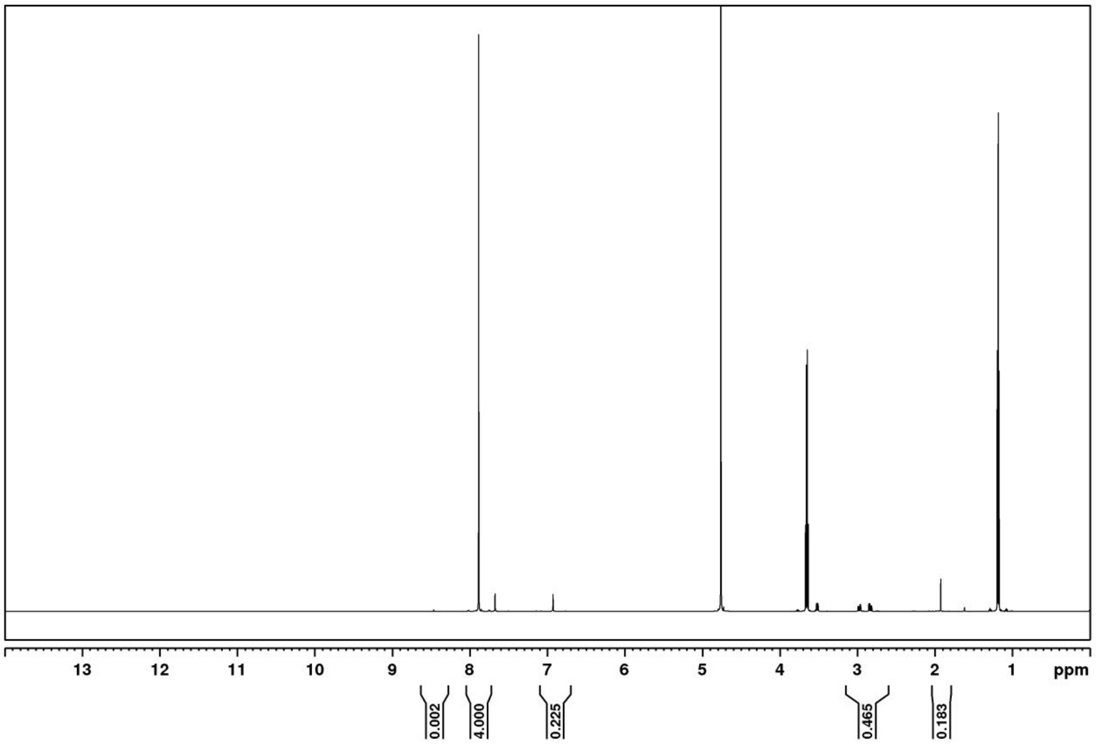

Figure S15 ^{1}H NMR spectrum (600 MHz, 1 M NaOD in D_2O) of UiO-66-his-10, replicate 2

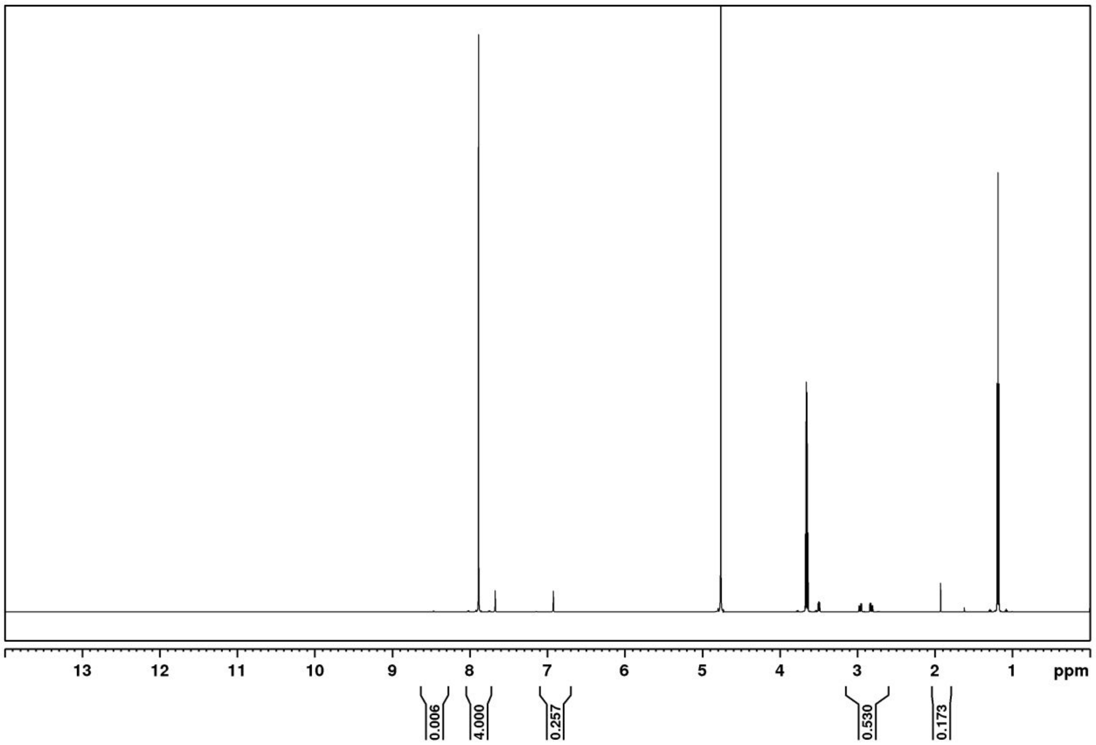

Figure S16 ^1H NMR spectrum (600 MHz, 1 M NaOD in D_2O) of UiO-66-his-10, replicate 3

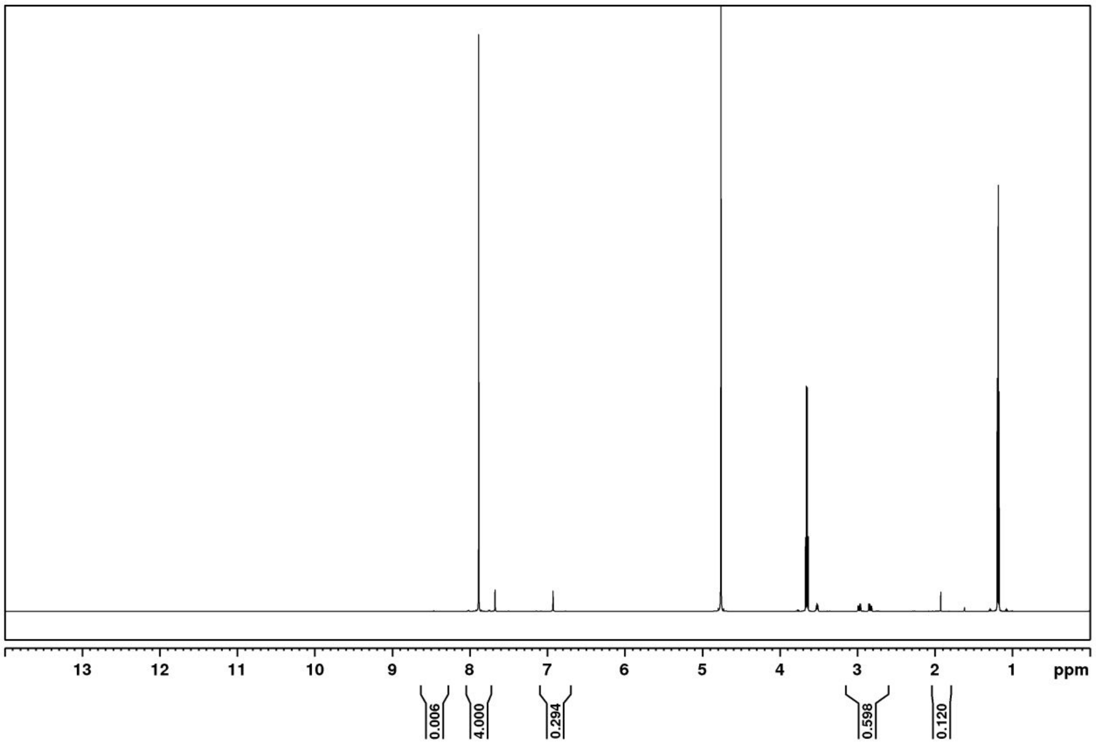

Figure S17 ^1H NMR spectrum (600 MHz, 1 M NaOD in D_2O) of UiO-66-his-15, replicate 1

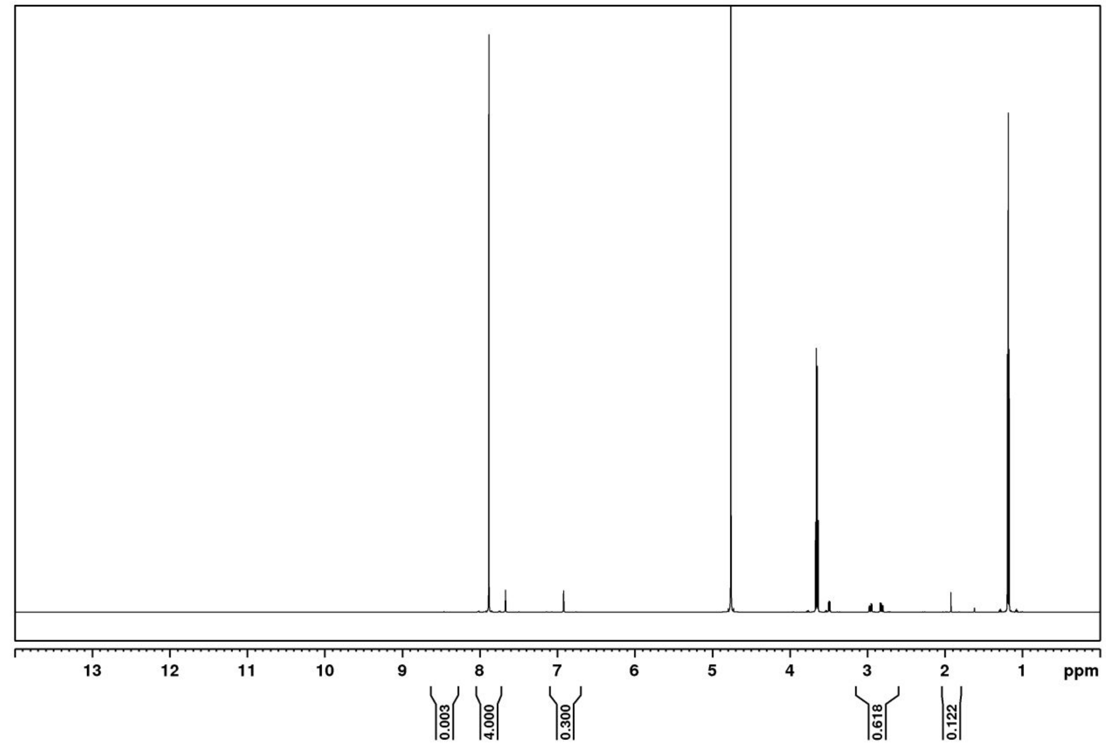

Figure S18 ^{1}H NMR spectrum (600 MHz, 1 M NaOD in D_2O) of UiO-66-his-15, replicate 2

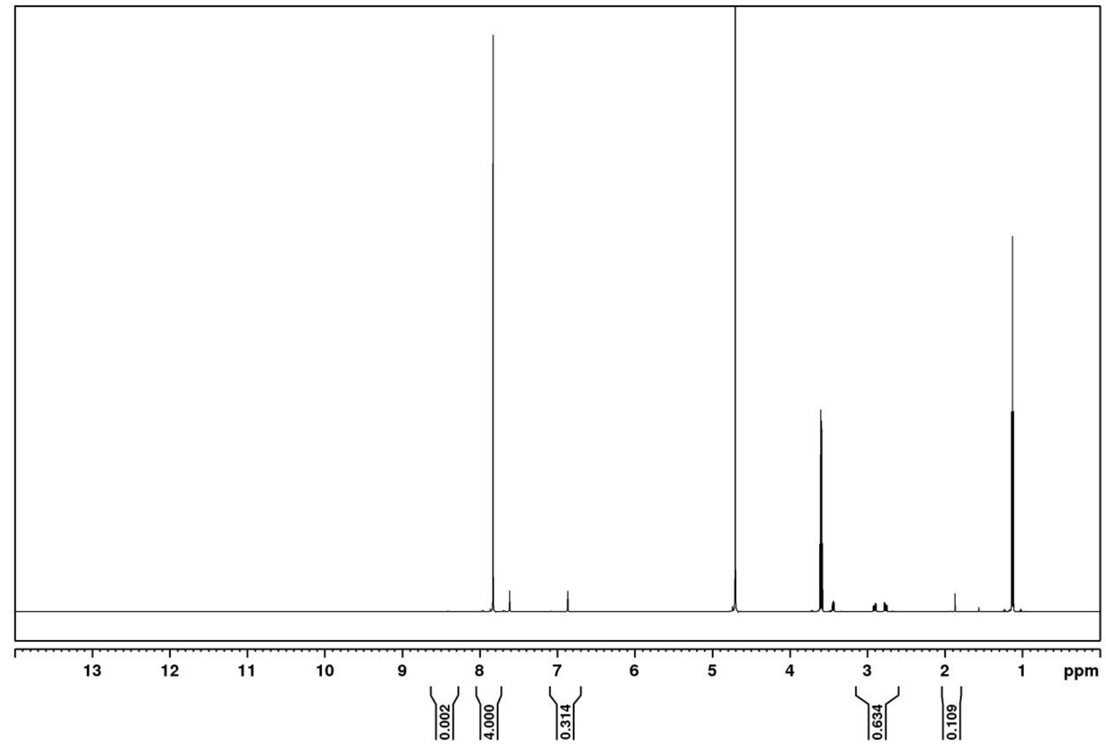

Figure S19 ^{1}H NMR spectrum (600 MHz, 1 M NaOD in D_2O) of UiO-66-his-15, replicate 3

Microwave-plasma atomic emission spectroscopy

Table S3: MP-AES results on digested UiO-66-his-X-Cu in Series 1.

Sample	Replicate	Cu^a/Zr_6^b	Average	Standard deviation (Relative)
**0**	1	0.04465	0.05	0.02 (31.8 %)
	2	0.07735		
	3	0.03865		
**1**	1	0.5602	0.54	0.01 (2.1 %)
	2	0.537		
	3	0.5342		
**2**	1	0.576	0.59	0.01 (1.4 %)
	2	0.5914		
	3	0.5944		
**3**	1	0.4796	0.42	0.05 (10.9 %)
	2	0.3886		
	3	0.3785		
**4**	1	0.3612	0.34	0.01 (4.2 %)
	2	0.3429		
	3	0.3258		
**5**	1	0.3092	0.30	0.01 (4.1 %)
	2	0.303		
	3	0.2808		

a: λ_{Cu} = 324.754 nm
b: λ_{Zr} = 343.823 nm

Table S4 Measured copper-to-zirconium ratios for UiO-66-his-X-Cu samples in Series 2A.

Histidine loading	Cu^a/Zr_6^b
**0**	0.20
**2.5**	1.18
**5**	0.95
**7.5**	0.90
**10**	0.66
**15**	0.55

a: λ_{Cu} = 324.754 nm
b: λ_{Zr} = 343.823 nm

Table S5 Measured copper-to-zirconium ratios for UiO-66-his-X-Cu samples in Series 2B.

Histidine loading	Cu^{a} / Zr_6^{b}
**0**	0.98
**2.5**	0.31
**5**	0.58
**7.5**	0.83
**10**	0.77
**15**	0.77

a: λ_{Cu} = 324.754 nm
b: λ_{Zr} = 343.823 nm

Thermogravimetric analysis

Thermograms for Series 1:

Average thermograms for Series 1 are shown in **Figure S20**, with their respective standard deviations are given as error bands. The individual thermograms are plotted in groups of three, starting from **Figure S21** through **Figure S26**.

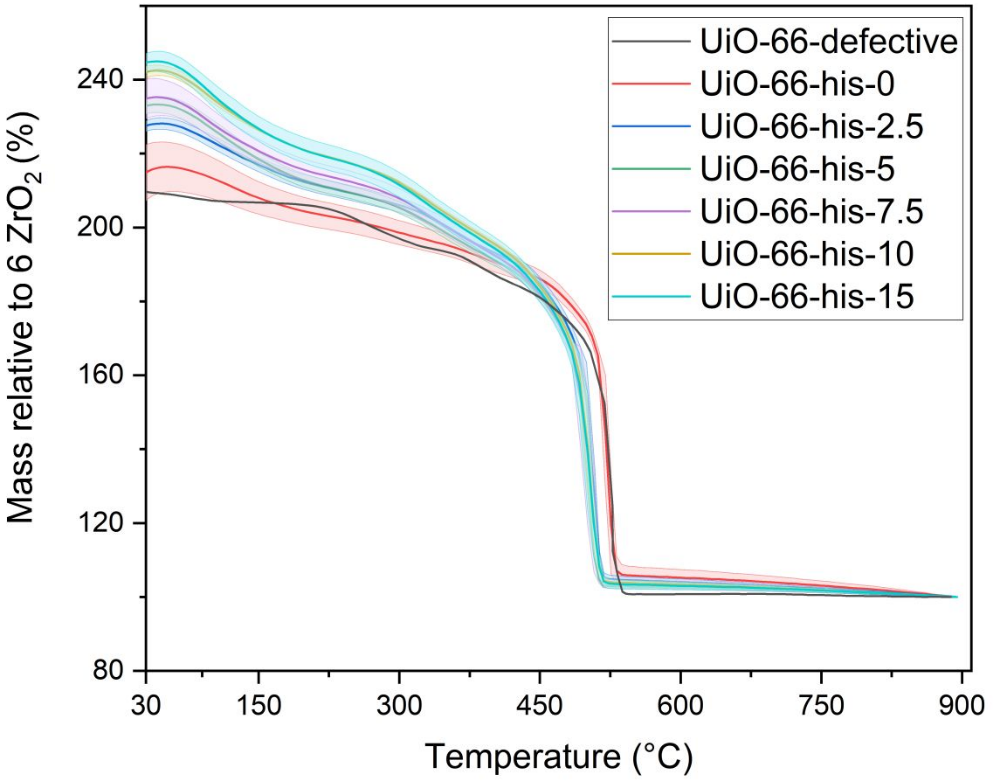

Figure S20: Thermogravimetric analysis of series 1, shown with error bands from triplicate samples.

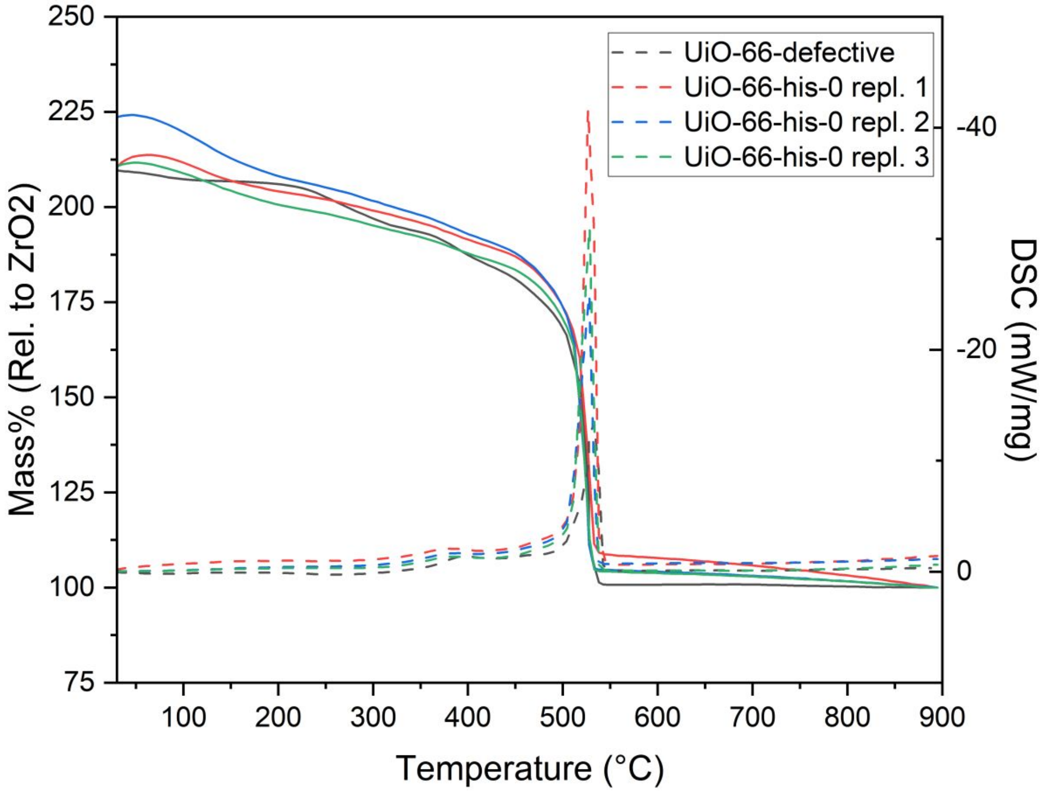

Figure S21 Thermogravimetric analysis of UiO-66-his-0. DSC signal is given as dashed lines.

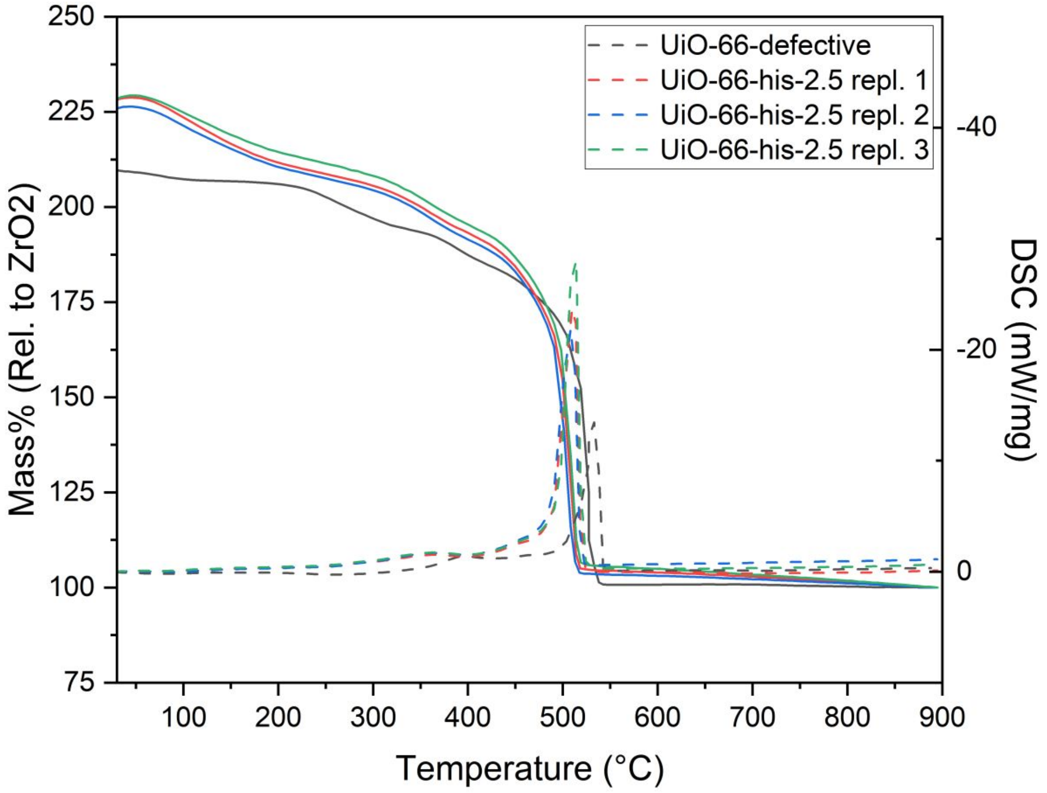

Figure S22 Thermogravimetric analysis of UiO-66-his-2.5. DSC signal is given as dashed lines.

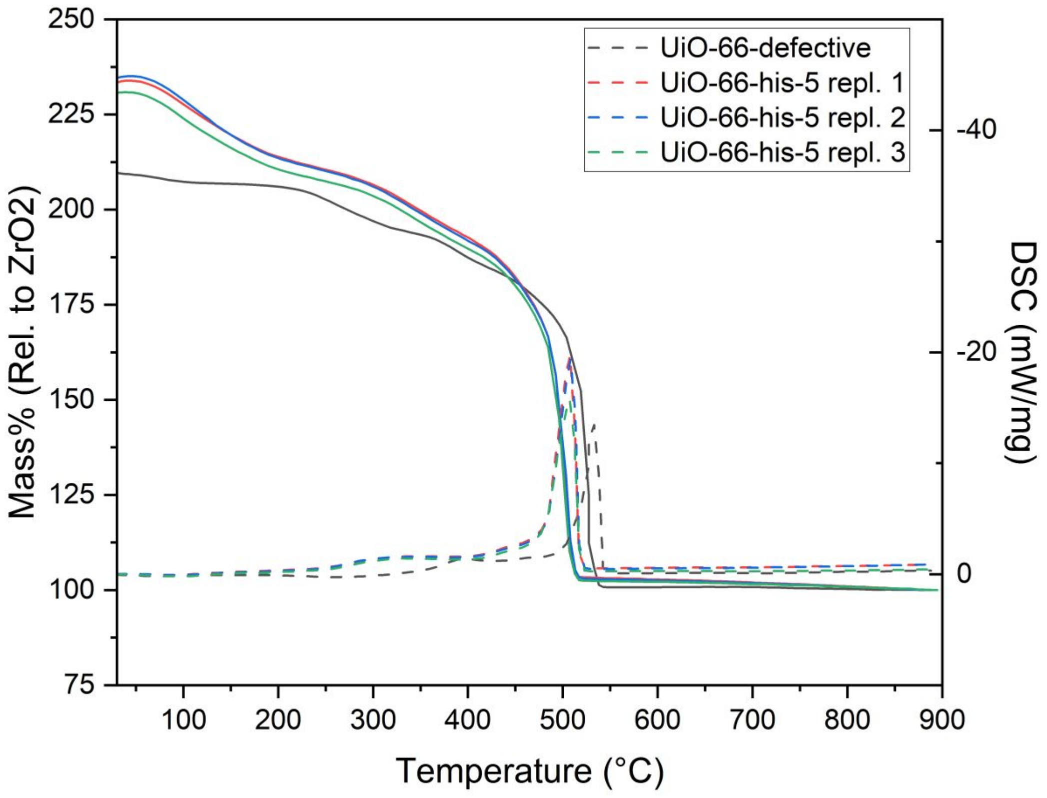

Figure S23 Thermogravimetric analysis of UiO-66-his-5. DSC signal is given as dashed lines.

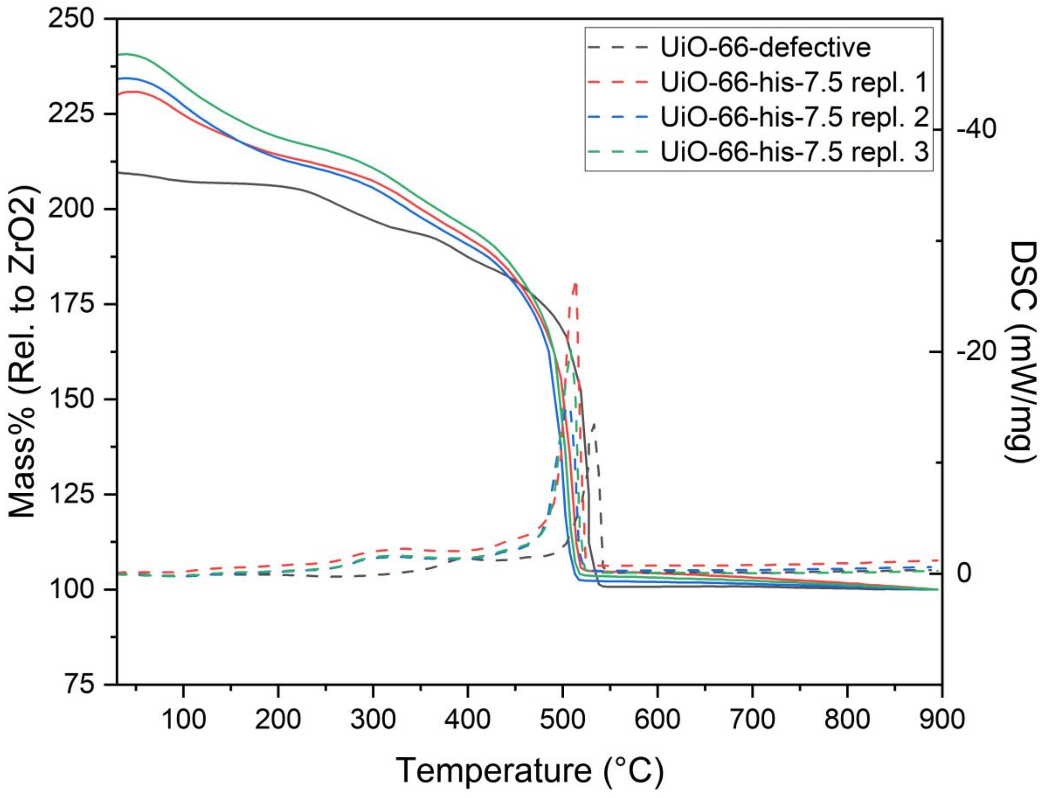

Figure S24 Thermogravimetric analysis of UiO-66-his-7.5. DSC signal is given as dashed lines.

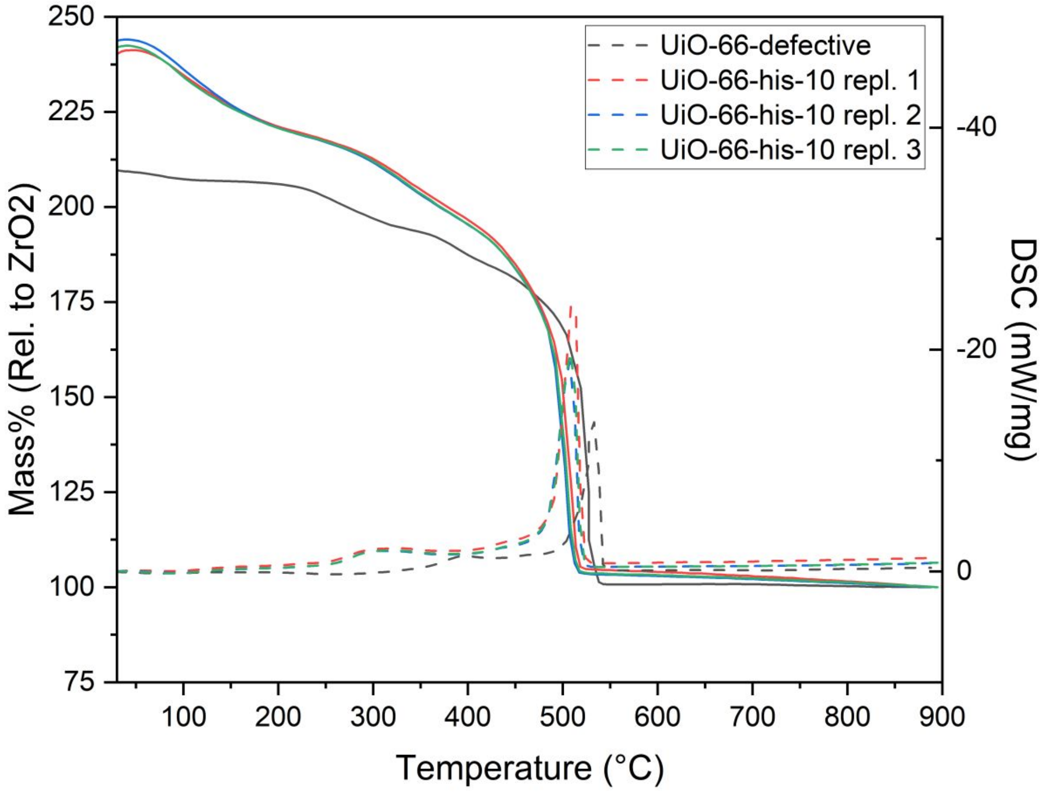

Figure S25 Thermogravimetric analysis of UiO-66-his-10. DSC signal is given as dashed lines.

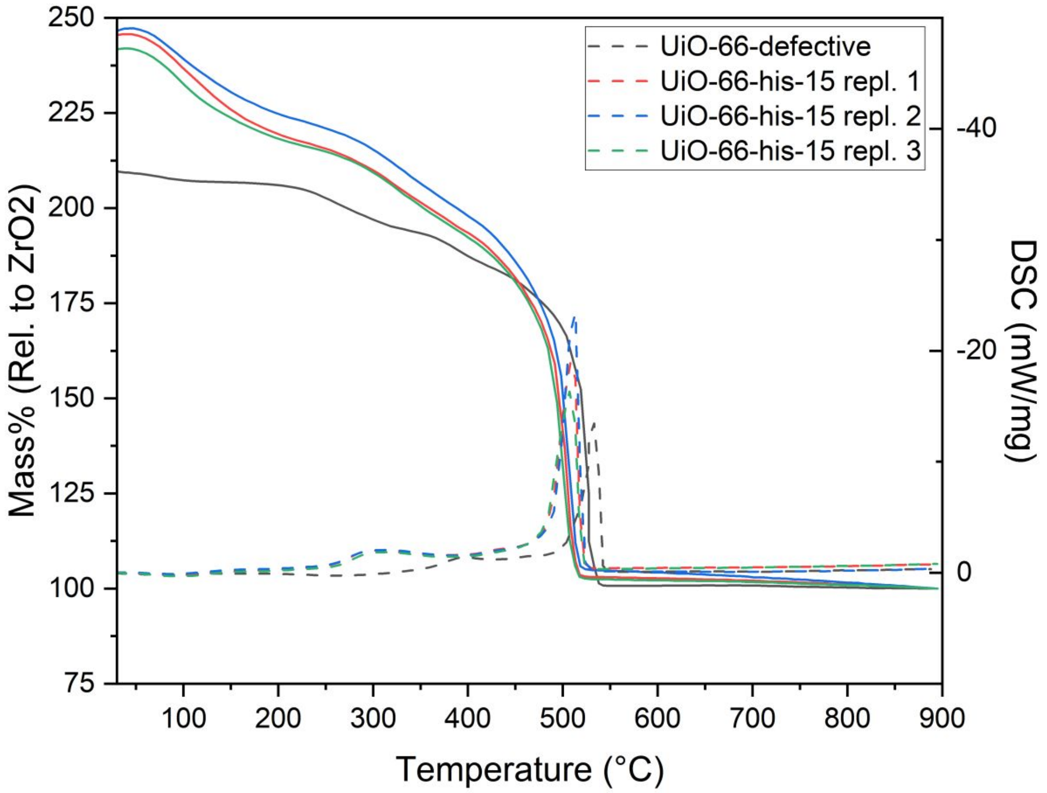

Figure S26 Thermogravimetric analysis of UiO-66-his-15. DSC signal is given as dashed lines.

Thermograms for Series 2

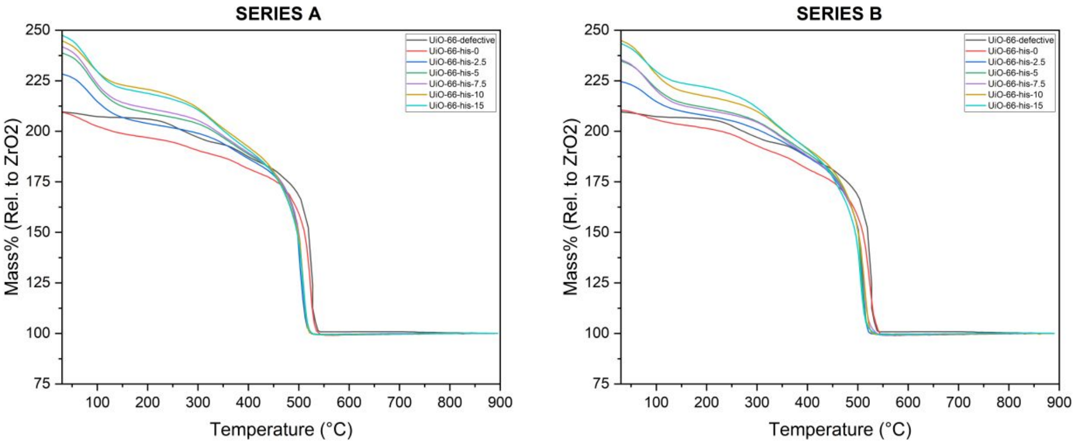

Figure S27 Thermogracimetric analysis of UiO-66-histidine for Series A (left) and Series B (right).

Compositional summary

Table S6: Averaged compositional data for UiO-66-his-X-Cu (series 1).

Sample	Histidine equiv.†	Incorporation, relative to Zr_6-cluster					
		Histidine	Copper	Acetate	Formate	H_2O/OH‡	Cu/His-ratio
0	0.0	-	0.05 ± 0.02	0.97 ± 0.05	0.65 ± 0.03	2.3 ± 0.5	-
1	2.5	0.30 ± 0.05	0.54 ± 0.01	0.85 ± 0.07	0.19 ± 0.02	1.6 ± 0.3	1.7
2	5.0	0.63 ± 0.06	0.59 ± 0.01	0.51 ± 0.02	0.069 ± 0.005	1.9 ± 0.2	0.94
3	7.5	0.99 ± 0.06	0.42 ± 0.05	0.34 ± 0.02	0.032 ± 0.004	1.6 ± 0.4	0.42
4	10	1.15 ± 0.06	0.34 ± 0.01	0.283 ± 0.009	0.017 ± 0.008	0.87 ± 0.04	0.29
5	15	1.43 ± 0.06	0.30 ± 0.01	0.18 ± 0.02	0.017 ± 0.008	1.0 ± 0.4	0.21

†: Molar equivalents to MOF with estimated molar mass of 1500 $gmol^{-1}$.

‡: Estimated with NMR/TGA, with the method described in[20]

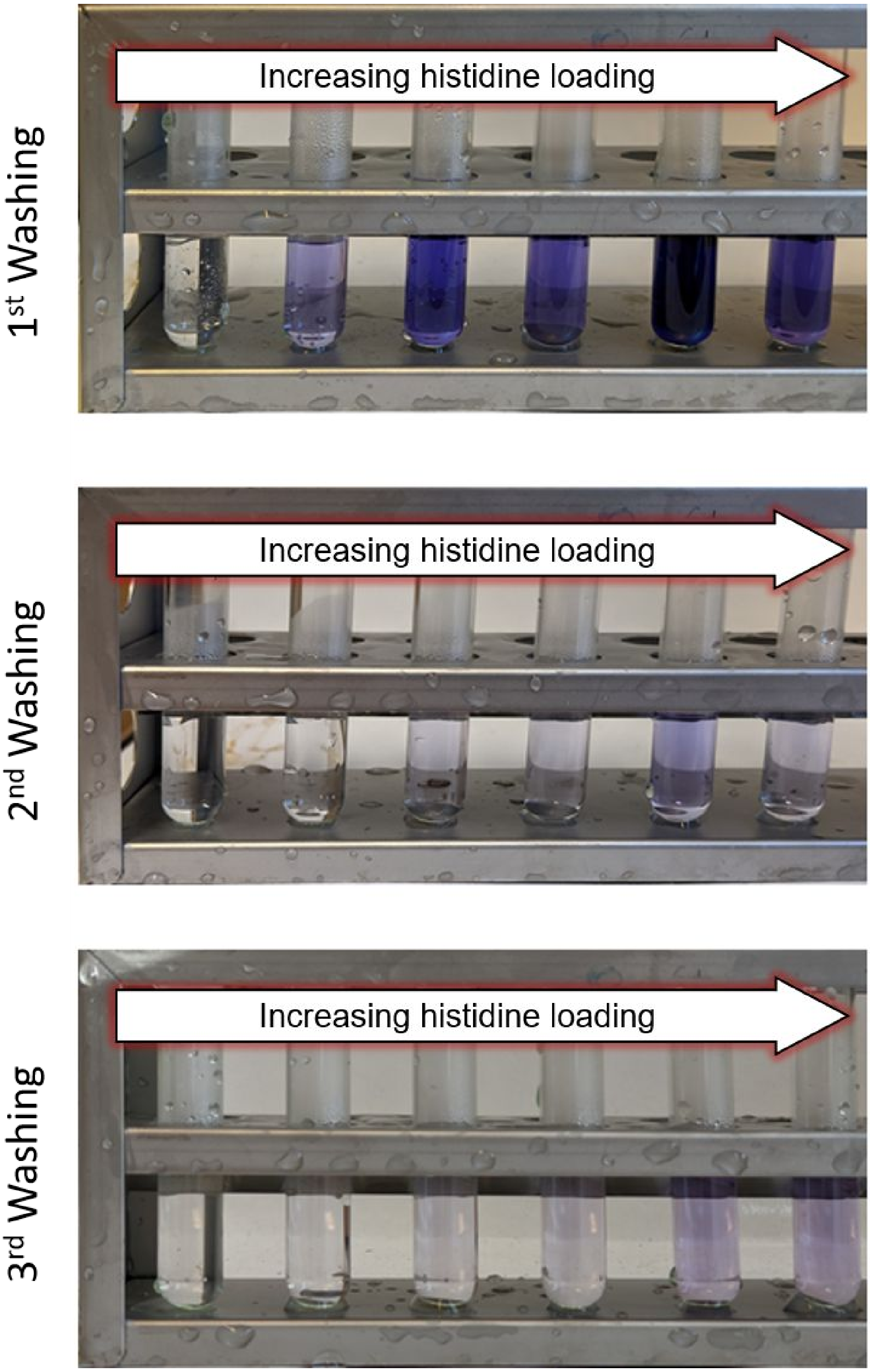

Figure S28: Kaiser's test of filtrate from washed histidine functionalized UiO-66. Samples of increasing histidine amount (left to right, leftmost being blank).

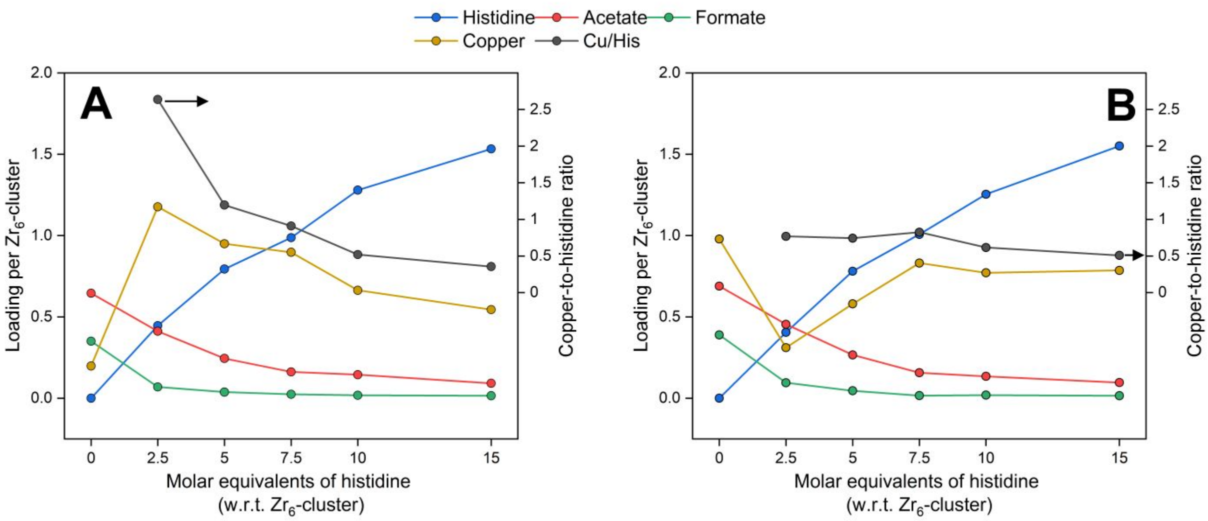

Figure S29 Compositional analysis of series 2A (Cu(I)I, left) and series 2B (Cu(II)$(BF_4)_2$, right).

Diffuse reflectance UV-VIS

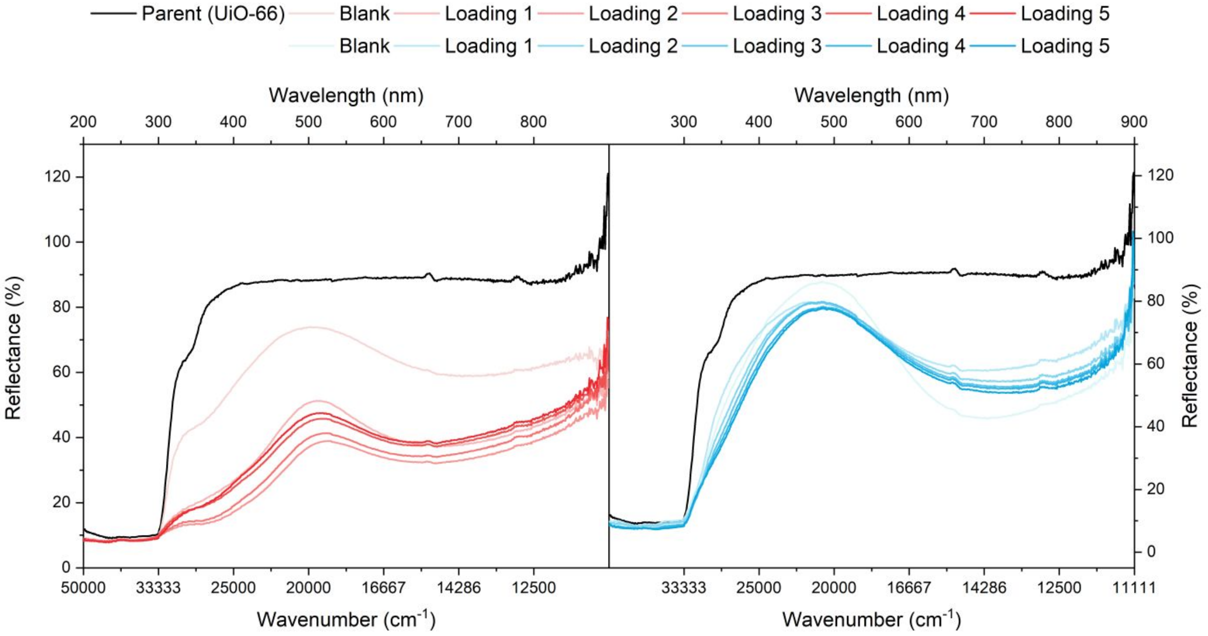

Figure S30: DR-UV-Vis spectra for series 2A (Cu(I)I), left) and series 2B (Cu(II)$(BF_4)_2$, right).

Powder X-ray diffraction

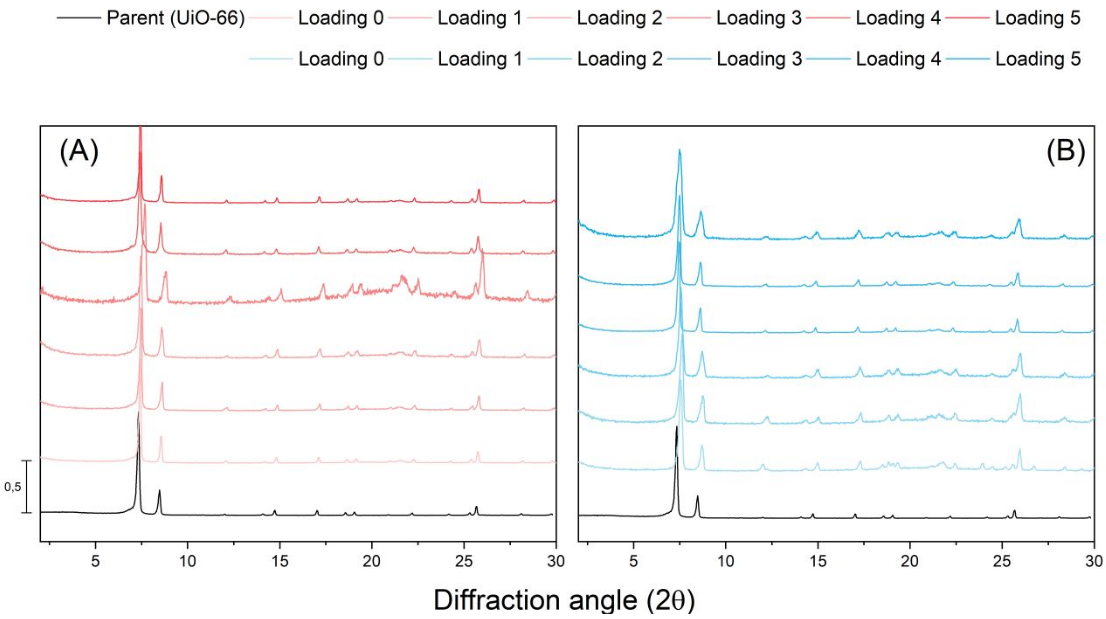

Figure S31: Powder X-ray diffraction patterns of series 2A and 2B (left and right).

EPR measurements

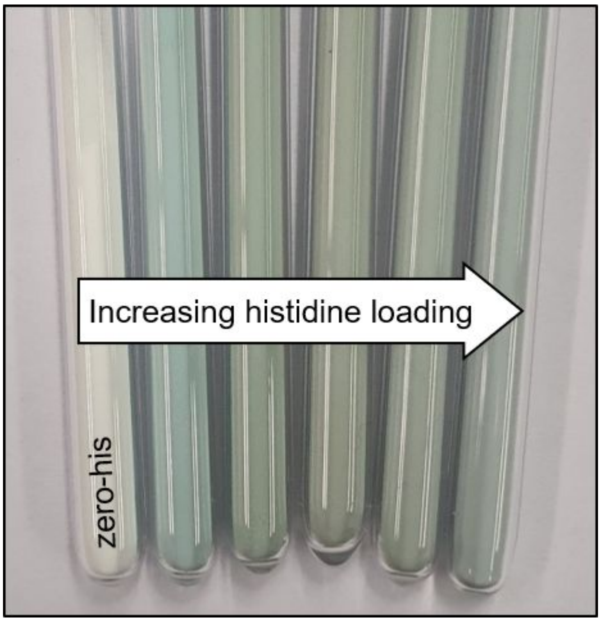

Figure S32: Photograph of series 1 loaded for EPR measurements. The histidine containing samples display a clear color change.
